# Latin American consensus on tapering of biological therapy in psoriasis^☆^

**DOI:** 10.1016/j.abd.2025.501134

**Published:** 2025-06-18

**Authors:** Angela María Londoño-García, Juan Raúl Castro-Ayarza, Manuel Darío Franco Franco, Cesar Fernando González Ardila, Gabriel Magariños, Enrique Salvador Rivas Zaldívar, Susan Martínez, Linda Ibatá, Julieth Carolina Castillo, Paola Jimena Cárdenas Rojas, Evelyn Giuliana Castro Vargas, Claudia Romina Contreras, Claudia de la Cruz Fernández, Andrés Chavarriaga Restrepo, Cristina Mariela Echeverria, André Vicente Esteves de Carvalho, Benjamín Hidalgo Matlock, Enrique Loayza, Matías Rafael Maskin, Ricardo Romiti, Fernando Valenzuela

**Affiliations:** a Dermatology Postgraduate Program, Faculty of Medicine, CES University, Medellin, Colombia; b Dermatology Postgraduate Program, Faculty of Medicine, National University of Colombia, Bogota, Colombia; c Dermatology Service, Medicarte IPS, Bogota, Colombia; d Private Practice, Bogota, Colombia; e Dermatology Service, Clinical Practice and Research, Dermatological Medical Center - Psoriahue, Buenos Aires, Argentina; f Research Department, Dermatological Medical Center DERMOS, Ciudad de Guatemala, Guatemala; g Epidemiology Department, EpiThink Health Consulting, Bogota, Colombia; h Dermatology Service, National Hospital Alberto Sabogal Sologuren, Callao, Peru; i Faculty of Medical Sciences, Hospital de Clinicas, National University of Asunción, Asuncion, Paraguay; j Dermatology Service, Clínica Dermacross, Santiago, Chile; k Rheumatology Service, CES Clinic, Medellin, Colombia; l Dermatology Service, Institute of Psychophysical Rehabilitation, Buenos Aires, Argentina; m Dermatology Service, Moinhos de Vento Hospital, Porto Alegre, Brazil; n Faculty of Medicine, University of Costa Rica, Latin University of Costa Rica, San Jose, Costa Rica; o Dermatology Service, Institute of Rheumatology, Hematology, and Dermatology (IRHED), Guayaquil, Ecuador; p Dermatology Service, CEMIC, Dermatology Service, Buenos Aires Skin, Buenos Aires, Argentina; q Departamento de Dermatología, Universidad de São Paulo, São Paulo, Brasil; r Departamento de Dermatología, Universidad de Chile, Departamento de Dermatología, Universidad de los Andes, Chile

**Keywords:** Biological therapy, Consensus, Delphi technique, Drug tapering, Latin America, Psoriasis

## Abstract

**Background:**

Biologic therapy is an effective psoriasis treatment. However, its long-term use carries the risk of serious adverse effects and high costs for the healthcare system, which is particularly relevant in resource-limited Latin American countries. Therefore, there is scientific interest in exploring the feasibility of tapering therapy in low disease activity or clinical remission.

**Objective:**

To provide expert consensus-based recommendations to guide personalized and efficient tapering of biologic agents in adult patients with cutaneous psoriasis.

**Methods:**

Following an exhaustive systematic literature review, a consensus was developed using a modified Delphi methodology by a group of Latin American clinical dermatologists and an independent methodological team. The topics covered include treatment goals, tapering objectives and strategies, regimens, monitoring and tapering failure, and implementation considerations in patients with psoriasis treated with biological agents.

**Results:**

The expert panel reached a consensus on five general principles and 13 recommendations for tapering biological therapy for psoriasis. These recommendations provide scientific support for dermatologists and healthcare providers regarding criteria for tapering, strategies and regimens, monitoring, failure management, and considerations for implementation.

**Conclusion:**

Tapering of biologics appears to be effective and safe in psoriasis patients with low stable activity or clinical remission. This Latin American consensus was developed in recognition of the need for rational and optimal use of biologics, while individualizing cases to apply best clinical practices.

## Introduction

Psoriasis is a chronic inflammatory skin disease with a strong genetic predisposition and autoimmune pathogenic features.^1^ It is estimated to affect between 2% and 3% of the world's population, with a higher incidence in Nordic countries and a lower incidence in equatorial countries.^1^ In Latin America and the Caribbean, the incidence of psoriasis has been estimated at 120 per 100,000 patient-years among those attending a dermatology consultation,^2^ representing a high burden on the healthcare system. Long-term therapy is often required for its management. The treatment choice is determined by disease severity, comorbidities, and access to medical care.^1^

When first-line treatments, such as methotrexate or cyclosporine, fail to control the disease or are contraindicated,^3^ biological therapy has proven to be effective in reducing skin lesions, joint involvement, inflammatory burden, and emerging comorbidities.^4^ Despite its efficacy, the prolonged use of biological agents is associated with serious adverse events and a high cost of care. Consequently, it is of scientific interest to analyze whether a gradual dose reduction can diminish the disadvantages of prolonged use of this therapy once a stable state of low disease activity or clinical remission is achieved.^4^

The evidence remains inconsistent regarding the dose tapering of biologics once the therapeutic goal is achieved. Gradual dose tapering has been shown to offer potential benefits by reducing side effects, the burden of repeated injections, and the cost of biological therapy.^4^ However, clinical guidelines for gradual dose tapering of biologics in patients with psoriasis are lacking, especially in low-income countries where the annual cost of this type of therapy can be double that of European countries.^5^, ^6^

The objective of this Latin American initiative is to provide recommendations based on expert consensus to guide the personalized and efficient management of dose reduction of biological agents in adult patients with cutaneous psoriasis, as well as to provide eligibility criteria, reduction strategies, and outcomes to be evaluated when deciding to carry out this strategy, guaranteeing the efficacy and safety of treatment.

This document is intended for dermatologists, especially those who use biological therapy in their clinical practice, and other health care and administrative professionals interested in the subject in the context of rational use of health care resources. The recommendations should be analyzed and implemented in the context of individualized care, adapted to individual clinical circumstances and the realities of Latin American countries.

## Methodology

### Participants

The consensus group comprised clinical dermatologists who discussed, defined, and voted on the final recommendations. They were selected from different countries in Latin America (Argentina, Brazil, Chile, Colombia, Costa Rica, Ecuador, Guatemala, Mexico, Paraguay, Peru) based on their specific experience with psoriasis and willingness to participate in related activities. An independent methodology team guided the entire process. All participants completed a declaration of interest, which was analyzed to identify potential conflicts. Information on the participants and their declarations of interest is described in Supplementary Appendix 1.

### Scope and evidence search

The target population is people aged 18-years and older diagnosed with cutaneous psoriasis, excluding arthropathy, who are being treated with biological agents and for whom tapering has been decided. For this document, tapering refers to the reduction in dose or frequency of the amount of biological medication administered to the patient once therapeutic goals have been achieved. Topics covered include treatment goals, tapering strategies, regimens, monitoring, tapering failure, and implementation considerations.

An exhaustive systematic literature review was performed to inform the recommendations for tapering biological therapy in psoriasis patients. Searches were conducted in electronic databases, including Medline (PubMed), Embase, and Ovid, as well as databases from compiling agencies and developers of clinical practice guidelines. Search strategies were tailored to each database using the following terms: “psoriasis” AND “tapering” OR “dose-adjustment” OR “optimization” AND “biologic”. Initial searches were conducted in May 2022 and updated in April 2023.

The inclusion criteria encompassed clinical trials, observational studies, and systematic literature reviews that evaluated the tapering or suspension of biological therapy. Secondary sources, such as clinical practice guidelines, evidence-based recommendation documents, and economic studies, were also considered based on information needs. Documents produced in the last seven years available as full publications, abstracts (provided they contained relevant information), in press, or gray literature were included. There were no language restrictions. Additionally, searches were conducted on the websites of relevant international scientific societies.

For the selection of identified references, two reviewers independently evaluated the documents based on the eligibility criteria. The initial screening was performed by reviewing titles and abstracts, followed by a full-text review. Disagreements regarding inclusions were resolved through discussion. For the quality assessment of the selected studies, the Cochrane risk of bias tool^7^ was used for Randomized Clinical Trials (RCTs),^7^ the Newcastle-Ottawa Scale^8^ for observational studies, and the Joanna Briggs Institute Checklist^9^ for analytical cross-sectional studies. Detailed specifications of the search, selection, and quality assessment processes are provided in Supplementary Material 1.

### Delphi process

A formal consensus was reached using the modified Delphi methodology. Based on the selected evidence, a list of statements was prepared according to a thematic matrix. The panel of experts reviewed the questionnaire to determine the need for adjustments, deletions, or additions. The final version of the questionnaire included 64 items organized into thematic blocks: criteria for tapering (goals and objectives), strategies and regimens, follow-up, definition of failure or relapse during tapering, re-treatment, and other considerations.

**Fig. 1:**
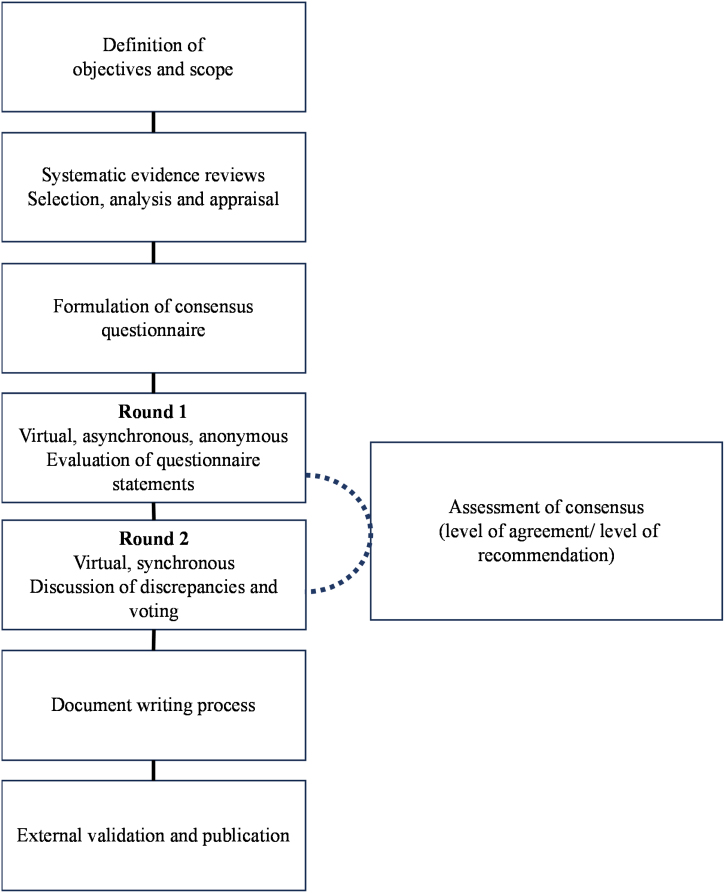
Consensus development methodology.

In the first round, all panel members completed the Delphi questionnaire asynchronously and anonymously online. The degree of agreement with the statements was recorded using a 5-point Likert scale.^10^ The expert judgments were tabulated and presented in a second round, maintaining the confidentiality of each member's opinions. Items lacking clear consensus and controversial aspects were addressed through exhaustive deliberations and anonymous synchronous votes to reach the final consensus. Detailed Delphi process specifications and results are provided in Supplementary Material 2 and illustrated in Fig. 1.

### Presentation and interpretation of consensus statements

In the following section, the statements are organized by subject. When a statement indicates that an action “must be performed”, it signifies that there is solid evidence of moderate or high quality supporting the efficacy and benefits of the recommended action. Additionally, there is a consensus among panel participants that this action should be performed in almost all cases. Conversely, when it is mentioned that an action “could be carried out” or “could be considered”, it implies that the intervention is supported by limited or low-quality evidence or lacks applicability to Latin American scenarios. The general agreement among the panel is that such actions could be implemented in selected circumstances, considering the particular characteristics of the patient and the context of care.

## Results and discussion

The consensus process resulted in 13 recommendations for tapering biological therapy in psoriasis patients. The use of strategies to reduce the dose or extend the interval of administration of biological agents in patients with psoriasis should be planned according to the general principles described in Table 1.

**Table 1:** General principles for tapering biologic therapy in psoriasis.

General principles
The decision to taper biologic agents in psoriasis must weigh benefits and risks through an individualized assessment of each patient.
Each patient evaluation should be based on disease severity, presence of comorbidities, quality of life, and patient values and preferences.
The statements generated from this consensus serve as suggested practice recommendations without constituting a restrictive care guideline for patients with psoriasis.
Particularly in the Latin American context, it is important to consider the realities of the specific clinical setting and the availability of resources when implementing the recommendations.

The characteristics of the selected studies are described in detail in Supplementary Appendix 3. This evidence includes eight clinical trials (10 publications) that evaluated the efficacy of dose reduction or interval prolongation of biological agents.^11^, ^12^, ^13^, ^14^, ^15^, ^16^, ^17^, ^18^, ^19^, ^20^ These studies included between 111 and 2,546 patients who had been treated for ≥12 or 24 weeks with agents such as adalimumab, etanercept, ustekinumab, brodalumab, secukinumab, and guselkumab. Seven clinical trials (eight publications)^13^, ^16^, ^19^, ^20^, ^21^, ^22^, ^23^, ^24^ evaluated the complete discontinuation of biological therapies such as etanercept, adalimumab, tildrakizumab, and risankizumab (n = 3,298 patients), with treatment duration ranging from 4-months to nearly 5-years before discontinuation. Furthermore, 22 observational studies investigated biologic tapering in psoriasis patients.^11^, ^25^, ^26^, ^27^, ^28^, ^29^, ^30^, ^31^, ^32^, ^33^, ^34^, ^35^, ^36^, ^37^, ^38^, ^39^, ^40^, ^41^, ^42^, ^43^, ^44^, ^45^ These studies included sample sizes ranging from 2 to 223 patients with treatments such as adalimumab, etanercept, ustekinumab, infliximab, guselkumab, ixekizumab, and secukinumab, with biological treatment durations of at least six months. Information from six secondary sources was also included: consensus documents,^46^, ^47^, ^48^ practice guidelines,^49^, ^50^ and recommendation documents.^51^

The evidence for each topic is described below.

### Criteria for tapering

Table 2 describes the consensus statements on criteria for tapering of biological therapy in psoriasis.

**Table 2:** Consensus statements on criteria for tapering biologic therapy in psoriasis.

Statements	Agreement level
Tapering of biological therapy may be considered in patients with psoriasis who achieve and maintain the therapeutic goal of a sustained PASI100, PASI/BSA/PGA 0, for at least one year.	100%
Tapering of biological therapy may be considered in patients with psoriasis who achieve and maintain an absolute PASI < 3 for at least one year, according to individual clinical characteristics and in consultation with the patient.	67%^a^
Tapering of biological therapy may be considered in patients with psoriasis involving specific areas with complete or almost complete improvement (PGA 0‒1 ) for at least one year.	87%
Tapering of biologic therapy may be considered in patients with mild psoriasis, good general health, a low initial PASI score, a good response to biologic treatment, and a short time to clinical remission.	88%
When considering tapering of biologic therapy, health-related quality of life should be taken into account, as it may deteriorate significantly during treatment interruption.	94%

PASI, Psoriasis Area Severity Index; PGA, Physician Global Assessment; BSA, Body Surface Area.

a Unanimous agreement was not possible.

Published studies have shown wide heterogeneity in scales and cutoffs to define the treatment goals and the appropriate time to taper biological therapy. Recent advances in biologics development have motivated the establishment of more stringent therapeutic targets. The evidence consistently suggests strategies for tapering in psoriasis patients with a complete or near-complete response, measured by a PGA 0‒1, a PASI90, or a PASI ≤ 2.^11^, ^14^, ^15^, ^17^^,^^18^, ^52^, ^53^, ^54^, ^55^, ^56^

The initiation of tapering with less demanding therapeutic goals than complete clearance has been controversial; however, there is evidence of acceptable outcomes in these settings. In the United States, tapering adalimumab in patients with a PASI score ≥ 50 resulted in the maintenance of response at 24-weeks.^17^, ^55^ Observational studies in Spain and Europe^34^, ^35^, ^37^ have shown sustained clinical responses in the majority of patients at 6- and 12-months after tapering of biologic therapy, mainly for adalimumab and etanercept, when they achieved responses of ≥ PASI50, ≥ PASI75 or PASI < 3 maintained over time.

Evidence from the region includes a cohort study in Colombia (n = 467) that evaluated the tapering of various biologics in patients with psoriasis who achieved a sustained response, including an absolute PASI < 3, and reported that 88% of patients maintained a clinical response after eight months of follow-up.^43^ The consensus panel extensively discussed the appropriateness of an absolute PASI < 3 as a criterion for considering tapering strategies. However, unanimous agreement was not possible due to the variability of cases, particularly in psoriasis of special localizations. The use of thresholds other than complete clearance in psoriasis patients is a decision that should be considered on an individual basis according to the clinical manifestations and in consultation with the patient.

In a survey of dermatologists worldwide (n = 57), nearly half of the physicians used a PASI ≤ 1 or ≤ 2, a BSA ≤ 1 or ≤ 2, or a PGA ≤ 1 as criteria for initiating tapering of biologic therapy, while a quarter indicated that they would consider dose reduction only in psoriasis-free patients (PASI/PGA0). Overall, 64.9% of patients were tapered after at least one year of treatment.^51^ This finding is consistent with studies showing that tapering of biologic therapy typically occurs after maintaining individualized treatment goals for at least 12-months.

Even though the DLQI is rarely used to define the start of tapering of biological therapy,^11^ factors associated with improved quality of life, such as reduction in psoriasis-related symptoms and treatment satisfaction, are often considered. The use of the DLQI is important as a Patient-Reported Outcome (PROM) in the comprehensive assessment of psoriasis, but it requires guidance for optimal use and easy understanding by patients. According to the panel, these limitations prevent the DLQI from being established as a necessary criterion for determining the tapering of biologics in patients with psoriasis. However, the importance of the patient's perspective in tapering has been documented in dermatology clinical practice worldwide.^51^ Therefore, clinicians are urged to include a comprehensive assessment of patients and their circumstances as part of shared and informed decision-making.

Based on the results of several previous clinical studies, discontinuing biologic therapy should be considered if the patient's condition can be maintained in near remission for 40- to 52-weeks.^50^ No long-term evidence beyond one year has been presented for any biological product using this strategy.^47^, ^50^

The studies that included biologic discontinuation as a strategy evaluated the achievement of goals using PASI scores of ≥ 50 or ≥ 75, PGA scores of ≤ 2, or sPGA scores 0‒1, showing wide variability.^13^, ^16^, ^19^, ^20^, ^21^, ^22^, ^23^, ^24^ However, the results of these studies support the continuation of biological therapy to maintain efficacy over time, given the high relapse rates and deterioration in health-related quality of life associated with discontinuation of therapy.^50^

### Tapering strategies and regimes

Table 3 describes the consensus statements on tapering strategies and regimens for biologic therapy in psoriasis.

**Table 3:** Consensus statements on tapering strategies and regimens for biological therapy in psoriasis.

Statements	Agreement level
In patients with psoriasis who have achieved and maintained a therapeutic response, tapering of biological therapy aims to maintain the established goal while using the lowest possible effective dose.	92%
Tapering strategies include gradually increasing the intervals between administrations and reducing the dose for those medications that allow it.	92%
Biologic dosage regimen should allow for flexible dose modification (increasing the interval or reducing the dose).	82%
The tapering regimen should be based on the indications for each biologic, its dosing intervals and dose adjustment options (see Table 4).	100%

The evidence supports the possibility of tapering biological therapy in psoriasis patients who achieve and maintain a therapeutic response. Strategies for tapering include extending the intervals between doses, reducing the dose while maintaining continuous treatment, and using the lowest effective dose for maintenance therapy.^47^ For some molecules, studies have demonstrated the safety of reducing the initial dose by up to 50% while maintaining efficacy.^3^ Extending dosing intervals is the most commonly used strategy in observational studies, with 60% to 100% of patients maintaining clinical responses of ≥ PASI75, ≥ PASI90, or even PASI100 at various follow-up periods, typically up to one year.^7^, ^11^, ^25^, ^30^^,^^31^, ^33^, ^36^, ^40^, ^41^, ^42^, ^45^

The different tapering regimens for each biologic are shown in Table 4.

**Table 4:** Tapering regimens for biological treatment of psoriasis.

Biological	Standard Dosage	Initial Tapering Regimen	Extended Tapering Regimen^a^
Adalimumab	40 mg every 2-weeks	40 mg every 4-weeks	Then, every 6-weeks
Etanercept	50 mg every week	50 mg every 14-days	Up to 21-day interval
Infliximab	3 or 5 mg/kg every 8-weeks	Do not taper due to associated risks; prefer switching	
Ustekinumab^b^	45 mg (≤100 kg) every 12-weeks	45 mg every 16-weeks	Up to 24-week interval
	90 mg (>100 kg) every 12-weeks	90 mg every 16-weeks	Up to 24-week interval
		45 mg every 12-weeks	45 mg every 16-weeks
Secukinumab^c^	300 mg every 4-weeks	300 mg every 6-weeks	Up to 8-week interval
		150 mg every 4-weeks	Up to 6-week interval
Guselkumab	100 mg every 8-weeks	100 mg every 12-weeks	Up to interval every 16 weeks
Risankizumab	150 mg every 12-weeks	150 mg every 16-weeks	Until interval every 24-weeks
Certolizumab pegol	200 mg every 2-weeks	200 mg every 4 weeks	200 mg every 6-weeks
Ixekizumab	80 mg every 4-weeks	80 mg every 6-weeks	80 mg every 8 weeks

a If remission persists after 6-months of reduced dose therapy.

b Efficacy was better maintained over time in the standard-dose group than in the tapering group.

c An every 6-week regimen may result in a less pronounced PASI 90 responses in some patient groups (women, patients aged 65- to 75-years, and those with prior treatment for psoriasis).

In general, administration intervals are prolonged within a fixed schedule, with the possibility of further adjustments to increase the time between doses if disease stability is maintained after 6-months of therapy. These strategies could be adapted to different groups of patients. For example, a study that evaluated the impact of prolonged treatment intervals and early intervention (≤ 2-years from symptom onset) with guselkumab demonstrated the possibility of tapering in super responders (PASI0 at weeks 20 and 28) while maintaining favorable PASI and DLQI measures over time has been demonstrated.^52^

Intermittent therapy has been considered in patients with a good response and those with a history of short exacerbations (lasting less than six months per year).^47^ Evidence from randomized clinical studies with withdrawal and retreatment periods has described for guselkumab a median time to loss of PASI90 response of 15.2 weeks (23 weeks after the last dose of guselkumab)^57^ and for ixekizumab, a median time to loss of response (PASI ≤ 50) of 20.4 weeks (143 days).^58^

A systematic literature review^59^ revealed evidence of the efficacy and safety of intermittent therapy with tumor necrosis factor inhibitors (adalimumab,^16^ certolizumab pegol,^60^ etanercept,^23^, ^37^, ^56^, ^61^^,^^62^ infliximab),^24^, ^63^ anti-interleukin 12/23 (ustekinumab),^64^, ^65^ anti-interleukin 23 (guselkumab)^57^ and anti-interleukin 17 (brodalumab,^54^, ^58^, ^66^ ixekizumab^58^ and secukinumab^12^). Studies have shown that 60% or more of patients on intermittent therapy achieve disease control (as defined in each study) after re-treatment, with the safety profiles of the biologics during re-treatment being as expected. The exception to these general findings was infliximab, which had the lowest rate of achievement of the efficacy outcome (25% and 38% in the two dosing groups evaluated) and a higher incidence of adverse reactions. The consensus panel considers that infliximab tapering may impose immunological risks for patients; therefore, switching to a different biologic is preferred.

Another strategy addressed in these studies is complete treatment interruption. Clinical trials with adalimumab, etanercept, and risankizumab have reported shorter maintenance times of clinical response, with subsequent worsening of PASI scores and health-related quality of life.^13^, ^16^, ^19^, ^20^, ^21^, ^22^, ^23^, ^24^

Finally, the decision on the tapering strategy and regimen of biologics in psoriasis treatment should be based on an individualized assessment of the clinical situation, considering the duration of treatment, the achievement of the proposed goals, the specific characteristics of the biologic agent, the patient's satisfaction with the treatment, and the resources available to comply with the recommendations.

### Monitoring and failure during tapering

Table 5 describes the consensus statements on monitoring and managing failures during tapering of biologic therapy in psoriasis.

**Table 5:** Consensus statements on follow-up and failure of tapering of biologic therapy in psoriasis.

Statements	Agreement level
Patients undergoing tapering of biological therapy should be followed up every 3-months, or sooner if there is a worsening of symptoms (skin or joint) or any other situation that warrants it.	100%
The identification of any of the following criteria at any time during follow-up is considered a failure of the tapering strategy:	
Absolute PASI >3	100%
PGA/BSA >1	93%
Unacceptable increase in disease activity as judged by the patient	100%
Change in morphology (e.g., psoriasis with pustules)	93%
Reactivation or appearance of lesions in specific areas	100%
Onset of joint symptoms	100%
In the event of a relapse with PASI ≥ 10 in a patient with psoriasis undergoing tapering of biologic therapy, the induction dose of the biologic should be restarted.	100%
In the event of a relapse with PASI < 10 in a patient with psoriasis undergoing tapering of biologic therapy, the immediately previous effective dose of biologic therapy should be reinstated.	100%

PASI, Psoriasis Area Severity Index; PGA, Physician Global Assessment; BSA, Body Surface Area.

Follow-up times in trials that reduced the dose or extended the interval between doses of the biological agent varied widely. In clinical trials, patients are usually evaluated every 4- to 12-weeks,^14^, ^15^, ^17^, ^18^^,^^52^, ^53^, ^54^, ^55^, ^56^, ^67^ including those who have interrupted the biologic.^13^, ^16^, ^19^, ^20^, ^21^, ^22^, ^23^, ^24^ In observational studies, tapering strategies have been monitored, usually every 12- to 16-weeks or at increasingly longer intervals, depending on the time of biologic interruption.^11^, ^25^, ^26^, ^27^, ^28^, ^29^, ^30^, ^31^, ^32^, ^33^, ^34^, ^35^, ^36^, ^37^, ^38^, ^39^, ^40^, ^41^, ^42^, ^43^ The Spanish Psoriasis Group of the Spanish Academy of Dermatology and Venereology (AEDV) recommends monitoring every 8- to 12-weeks during the maintenance phase for the majority of biological agents, the maintenance phase was defined as the period of treatment starting after the induction phase, regardless of its length, based on the patient’s needs and it can last from several weeks to years, or even a lifetime, depending on the patient’s needs.^47^

The expert panel of this consensus considers that follow-up every three months is indicated for the Latin American context, considering the clinical circumstances and the different insurance scenarios and healthcare systems in the region.

To our knowledge, there is no consensus on the definition of biologic tapering failure. In general, a decrease in the PASI score (<50 or <75 or a decrease ≥50% from the improvement achieved prior to discontinuation), a PGA score ≥3 or loss of response status, or a DLQI score > 5 has indicated tapering failure in psoriasis patients in clinical trials.^14^, ^15^, ^17^, ^18^^,^^52^, ^53^, ^54^, ^55^, ^56^, ^67^ In observational studies, tapering failure was defined as loss of response, individually defined and measured by PASI or DLQI, between 3- and 24-months after the start of the strategy.^11^, ^25^, ^26^, ^27^, ^28^, ^29^, ^30^, ^31^, ^32^, ^33^, ^34^, ^35^, ^36^, ^37^, ^38^, ^39^, ^40^, ^41^, ^42^, ^43^ In trials where biological treatment was interrupted completely, failure was defined as a decrease in PASI score >50% from the initial improvement or a PGA score of ≥ 3.^13^, ^16^, ^19^, ^20^, ^21^, ^22^, ^23^, ^24^ The Spanish consensus on the evaluation and treatment of moderate-to-severe psoriasis defined relapse after discontinuation of an effective drug as a loss of ≥ 50% of the improvement achieved or a PGA score of >2 if a PGA 0‒1 was achieved at any time.^47^

In the opinion of this expert panel, the heterogeneity in the definitions of biologic tapering failure in psoriasis patients is a consequence of the variability in the definition of the goals of the strategy. In this context, any evidence of regression in achieving the treatment goals set for tapering indicates failure. Similarly, specific clinical circumstances, such as changes in the morphology of psoriasis (e.g., generalized erythrodermic or pustular psoriasis), reactivation or appearance of lesions in specific areas, and negative patient-reported events, such as the appearance of joint symptoms or an unacceptable increase in disease activity, must also be considered as tapering failures and require expedited evaluation to determine the appropriate course of action.

In the trials that reported relapse, less than 18% of patients who underwent tapering experienced relapse within 6-months to 40-weeks.^13^, ^17^, ^19^, ^55^ Times to relapse varied and no significant differences in relapse rates were reported according to the type of biological agent.^43^ Once loss of response has been established, therapeutic options include restarting biological therapy at the last effective dose, re-treatment with standard doses, rescue therapy or switching biological agents.^11^, ^25^, ^26^, ^27^, ^28^, ^29^, ^30^, ^31^, ^32^, ^33^, ^34^, ^35^, ^36^, ^37^, ^38^, ^39^, ^40^, ^41^, ^42^, ^43^ According to the consensus panel, the therapeutic decision in these patients should be based on the severity of the relapse. In the studies that reported this finding, most patients achieved a target PASI within 4^56^ to 12-weeks^17^, ^55^ of treatment restart. Recovery of these responses after re-treatment has also been reported in uncontrolled follow-up cohort studies.^31^, ^36^, ^45^ These findings further support the efficacy and safety of tapering biologic therapy in psoriasis patients.

### Considerations for implementation

Tapering strategies aim to minimize chronic immunomodulation, which is associated with opportunistic infections and other complications. In addition, tapering reduces the occurrence of side effects, and the anxiety associated with lifelong medication. Tapering is in line with the patient's preference to reduce the frequency of doses and the time spent on treatment. Strategies to reduce biological therapy in psoriasis patients also support technical-scientific rationalization within regional healthcare systems and address the general resource limitations in Latin America. Dermatology groups have reported cost savings as one of the main outcomes of reducing the dose of biologic agents.^51^ Cost studies have shown that dose reduction or prolongation of the administration interval, as well as intermittent therapy regimens, are viable alternatives to optimize biologic treatment and reduce therapy costs.^28^, ^46^

This document is based on the best available evidence at the time of its production. However, it is recognized that the results of ongoing studies or future research may require modifying some of the recommendations described here or generating new recommendations. The biologics addressed in the consensus are currently available or may soon be available in Latin American countries. Some molecules for which there is evidence to support tapering, such as brodalumab,^54^, ^58^, ^66^ were not included in specific recommendations because they are not available in Latin American countries. There are currently very few reports on tapering molecules such as risankizumab, so specific guidelines for these should be developed in future updates of this consensus.

The guidance provided in this consensus is intended to support the development of efficient and safe tapering strategies for biologics, but it is not a rigid guideline. As previously emphasized, flexibility in biologic tapering regimens must be maintained through detailed and individualized clinical assessments, considering patients’ values, preferences and circumstances in the context of shared decision-making. In this context, it is expected that physician-prescribed strategies for tapering or reinitiation of biologic therapy for psoriasis will be administratively facilitated to promote adherence, ensure timely follow-up, and improve the chances of maintaining stable disease with reduced pharmacological requirements.

## Conclusions

Tapering of biological therapies appears to be effective and safe in psoriasis patients with low stable activity or clinical remission. This Latin American consensus was developed in recognition of the need for rational and optimal use of such therapies, including both their administration and discontinuation, to maintain the best health outcomes for patients. This consensus aims to facilitate decision-making by incorporating the best and most recent available evidence, especially in heterogeneous populations with limited healthcare resources. Encouraging local research is essential to advance knowledge on issues related to biologic suspension and to inform future tapering updates.

## External review and consensus update

A preliminary version of this manuscript, previously approved by the authors, was submitted for external peer review. The need to update this consensus should be evaluated in three years or sooner if necessary.

## Financial support

This consensus is endorsed by the Latin American Psoriasis Society (SOLAPSO) and the Colombian Group of Psoriasis and Immunodermatology (COLPSOR), affiliated with the Colombian Society of Dermatology (ASOCOLDERMA). It was developed thoroughly and independently, with transparency and impartiality. The funders did not participate in the development of the consensus, the decisions of the panel, or the final manuscript.

## Authors’ contributions

Angela María Londoño García: Conception and design, analysis and interpretation of data, critical review of content, and final approval of the manuscript.

Juan Raúl Castro-Ayarza: Conception and design, analysis and interpretation of data, critical review of content, and final approval of the manuscript.

Manuel Darío Franco: Conception and design, analysis and interpretation of data, critical review of content, and final approval of the manuscript.

Cesar Fernando González Ardila: Conception and design, analysis and interpretation of data, critical review of content, and final approval of the manuscript.

Gabriel Magariños: Conception and design, analysis and interpretation of data, critical review of content, and final approval of the manuscript.

Enrique Salvador Rivas Zaldívar: Conception and design, analysis and interpretation of data, critical review of content, and final approval of the manuscript.

Susan Martínez: Contributed to the critical review of the literature, writing the initial version of the manuscript, editorial review of the final manuscript, conception and design, analysis and interpretation of data, critical review of content, and final approval of the manuscript.

Linda Ibatá: Contributed to the critical review of the literature, writing the initial version of the manuscript, editorial review of the final manuscript, conception and design, analysis and interpretation of data, critical review of content, and final approval of the manuscript.

Julieth Carolina Castillo: Contributed to the critical review of the literature, writing the initial version of the manuscript, editorial review of the final manuscript, conception and design, analysis and interpretation of data, critical review of content, and final approval of the manuscript.

Paola Jimena Cárdenas Rojas: Conception and design, analysis and interpretation of data, critical review of content, and final approval of the manuscript.

Evelyn Giuliana Castro Vargas: Conception and design, analysis and interpretation of data, critical review of content, and final approval of the manuscript.

Claudia Romina Contreras: Conception and design, analysis and interpretation of data, critical review of content, and final approval of the manuscript.

Claudia de la Cruz Fernández: conception and design, analysis and interpretation of data, critical review of content, and final approval of the manuscript.

Andrés Chavarriaga Restrepo: Conception and design, analysis and interpretation of data, critical review of content, and final approval of the manuscript.

Cristina Mariela Echeverria: Conception and design, analysis and interpretation of data, critical review of content, and final approval of the manuscript.

André Vicente Esteves de Carvalho: Conception and design, analysis and interpretation of data, critical review of content, and final approval of the manuscript.

Benjamín Hidalgo-Matlock: Conception and design, analysis and interpretation of data, critical review of content, and final approval of the manuscript.

Enrique Fabian Loaiza Sánchez: Conception and design, analysis and interpretation of data, critical review of content, and final approval of the manuscript.

Matías Rafael Maskin: Conception and design, analysis and interpretation of data, critical review of content, and final approval of the manuscript.

Ricardo Romiti: Conception and design, analysis and interpretation of data, critical review of content, and final approval of the manuscript.

Fernando Valenzuela: Conception and design, analysis and interpretation of data, critical review of content, and final approval of the manuscript.

## Conflicts of interest

### Developer group

Ángela María Londoño García: Dermatologist. Master of Epidemiology, Master of Autoimmunity. Postgraduate Dermatology Coordinator, CES University, Colombia. Latin American Psoriasis Society ‒ SOLAPSO, Colombian Psoriasis and Immunodermatology Group ‒ COLPSOR. Has been a speaker for: Abbvie, Boehringer Ingerhaim, Bristol, Eli Lilly, Jannsen, Novartis, Pfizer.

Juan Raúl Castro Ayarza: Dermatologist. Master of Oncological Dermatology. Candidate for a Master's Degree in Epidemiology. Professor at the National University of Colombia, Colombia. Latin American Psoriasis Society ‒ SOLAPSO, Colombian Psoriasis and Immunodermatology Group ‒ COLPSOR. He has been a speaker for AbbVie, Amgen, Eli Lilly, Janssen, Novartis, Pfizer.

Manuel Darío Franco Franco: Dermatologist. Founding Member of the Colombian Psoriasis and Immunodermatology Group ‒ COLPSOR, Colombia. Latin American Psoriasis Society ‒ SOLAPSO. He has been a speaker for AbbVie, Amgen, Eli Lilly, Janssen, Novartis, Pharmalab, and Sanofi.

César Fernando González Ardila: Dermatologist. Private Practice, Colombia. Latin American Psoriasis Society ‒ SOLAPSO, Colombian Psoriasis and Immunodermatology Group ‒ COLPSOR.

Gabriel Magariños: Dermatologist. Psoriahue, Argentina. Latin American Psoriasis Society ‒ SOLAPSO, Colombian Psoriasis and Immunodermatology Group ‒ COLPSOR. He has been a speaker for: AbbVie, Boehringer Ingelheim, Eli Lilly, Jannsen, and Novartis.

Enrique Salvador Rivas Zaldívar: Dermatologist. Fellowship in Dermatological Surgery. Doctor of Medicine, Guatemala. Latin American Psoriasis Society ‒ SOLAPSO, Colombian Psoriasis and Immunodermatology Group ‒ COLPSOR. He has been a speaker for AbbVie and Novartis.

### Panel of experts

Paola Jimena Cárdenas Rojas. Dermatologist. Master en Dermatología Oncológica. Master en Salud Publica. Colombia. Latin American Psoriasis Society ‒ SOLAPSO, Colombian Psoriasis and Immunodermatology Group ‒ COLPSOR. He has been a lecturer for AbbVie, Amgen, Elli Lilly and Janssen.

Evelyn Giuliana Castro Vargas. Dermatologist. Resident dermatology tutor at the University of San Martin de Porres, Perú. Latin American Psoriasis Society ‒ SOLAPSO. He has been a lecturer for AbbVie, Janssen and Tecnofarma.

Claudia Romina Contreras. Dermatologist. Master in Autoimmune Diseases. Paraguay. Latin American Psoriasis Society ‒ SOLAPSO.

Claudia de la Cruz Fernández. Dermatologist. Chile. Latin American Psoriasis Society ‒ SOLAPSO. Has been a speaker or researcher for AbbVie, Boehringer Ingelheim, Bristol Myers Squibb, Eli Lilly, Janssen, Novartis, Pfizer, Sandoz, UCB Pharma.

Andrés Chavarriaga Restrepo. Internist and rheumatologist. Colombia. Latin American Psoriasis Society ‒ SOLAPSO, Colombian Psoriasis and Immunodermatology Group – COLPSOR. He has been a lecturer for Amgen, Eli Lilly, Jansen, Novartis, Pfizer, Pharmalab.

Cristina Mariela Echeverria. Dermatologist. Argentina. Latin American Psoriasis Society ‒ SOLAPSO. He has been a lecturer at AbbVie, Boehringer Ingelheim, Bristol Myers Squibb, Eli Lilly, Jannsen, L'Oreal, Novartis, Pfizer, Sandoz, UCB Pharma.

Andrés Vicente Esteves de Carvalho. Dermatologist. Brasil. Latin American Psoriasis Society ‒ SOLAPSO. He has been a lecturer for AbbVie, Boehringer, Eli Lilly, Jansen and Novartis.

Benjamín Hidalgo Matlock. Dermatologist. Costa Rica. Latin American Psoriasis Society ‒ SOLAPSO. He has been a researcher for Cutera and for Novartis.

Enrique Fabian Loaiza Sánchez. Dermatologist. Dermatopathologist. Master in Clinical and Epidemiological Research. Catholic University Professor. Ecuador. Latin American Psoriasis Society ‒ SOLAPSO. He has been a lecturer for Janssen, Medicament and Novartis.

Matías Rafael Maskin. Internista. Dermatologist. Argentina. Latin American Psoriasis Society - SOLAPSO.

Ricardo Romiti. Dermatologist. Brasil. Latin American Psoriasis Society ‒ SOLAPSO. He has been a speaker for AbbVie, Boehringer, Eli Lilly, Janssen, LEO Pharma, Novartis, Teva and UCB.

Fernando Valenzuela. Dermatologist. Professor at the University of Chile. Chile. Latin American Psoriasis Society ‒ SOLAPSO. He has been a speaker for AbbVie, Boehringer Ingelheim, Eli Lilly, Jannsen, LEO, and Novartis.

### Methodological Team

Susan Martínez R. Physician, specialist in Epidemiology, Master in Public Health. Epithink Health Consulting.

Linda Ibatá. Physician, specialist in Epidemiology, Master in Public Health. Epithink Health Consulting.

Julieth Carolina Castillo. RN, Specialist in Epidemiology, Master in Public Health. Epithink Health Consulting.
