# Supplementary material for: Latin American consensus on tapering of biological therapy in psoriasis
Source: An Bras Dermatol. 2025 Jun 18;100(4):501134. doi: 10.1016/j.abd.2025.501134 (PMC12213954; doi:10.1016/j.abd.2025.501134)
Supplement: Supplementary file 1 [file mmc1.docx]

ABD-D-24-00514

**Supplementary material**

**Supplementary material 1. Search, selection, and quality of the evidence**

# Evidence searches strategies

**Database, Platform: Medline, Pubmed**

**Date of search: May 2022**

**Search strategy (results)**

1 "psoriasis"[MeSH Terms] 45,99
2 "adjust*"[All Fields] 773,357
3 "de-escalation"[All Fields] 2,963
4 "discontinu*"[All Fields] 144,999
5 "dose"[All Fields] AND ("adaptation, psychological"[MeSH Terms] OR ("adaptation"[All Fields] AND "psychological"[All Fields]) OR "psychological adaptation"[All Fields] OR "adjustment"[All Fields] OR "adjust"[All Fields] OR "adjusted"[All Fields] OR "adjusting"[All Fields] OR "adjustments"[All Fields] OR "adjusts"[All Fields]) 48,527
6 "drug tapering"[MeSH Terms] OR ("drug"[All Fields] AND "tapering"[All Fields]) OR "drug tapering"[All Fields] OR ("dose"[All Fields] AND "reduction"[All Fields]) OR "dose reduction"[All Fields] 122,802
7 "dose"[All Fields] AND ("spaced"[All Fields] OR "spacing"[All Fields] OR "spacings"[All Fields]) 1,659
8 "drug tapering"[MeSH Terms] OR ("drug"[All Fields] AND "tapering"[All Fields]) OR "drug tapering"[All Fields] OR ("dose"[All Fields] AND "tapering"[All Fields]) OR "dose tapering"[All Fields] 4,477
9 "dose-adjustment"[All Fields] AND ("strategie"[All Fields] OR "strategies"[All Fields] OR "strategy"[All Fields] OR "strategy s"[All Fields]) 431
10 ("dosed"[All Fields] OR "doses"[All Fields] OR "dosing"[All Fields] OR "dosings"[All Fields]) AND "down"[All Fields] 7,676
11 "down"[All Fields] AND "titrat*"[All Fields] 1,041
12 "drug dosage calculations"[MeSH Terms] 2,75
13 "drug tapering"[MeSH Terms] 320
14 "escalat*"[All Fields] 44,978
15 "interrupt*"[All Fields] 82,352
16 "optimis*"[All Fields] 64,073
17 "optimiz*"[All Fields] 478,713
18 "re escalat*"[All Fields] 76
19 "restart*"[All Fields] 7,437
20 "spare"[All Fields] OR "spared"[All Fields] OR "spares"[All Fields] OR "sparing"[All Fields] 73,223
21 "stop*"[All Fields] 167,677
22 "taper*"[All Fields] 27,286
23 "taper"[All Fields] OR "tapered"[All Fields] OR "tapering"[All Fields] OR "tapers"[All Fields] 25,175
24 ("therapeutics"[MeSH Terms] OR "therapeutics"[All Fields] OR "treatments"[All Fields] OR "therapy"[MeSH Subheading] OR "therapy"[All Fields] OR "treatment"[All Fields] OR "treatment s"[All Fields]) AND ("withdraw"[All Fields] OR "withdrawal"[All Fields] OR "withdrawals"[All Fields] OR "withdrawing"[All Fields] OR "withdraws"[All Fields]) 81,114
25 "withdraw*"[All Fields] 148,499
26 "withhold*"[All Fields] 20,337
27 "Retreatment"[MeSH Terms] 10,409
28 "re‐treat*"[All Fields] 3,538
29 "bdmard"[All Fields] OR "bdmards"[All Fields] 1,099
30 ("biological products"[MeSH Terms] OR ("biological"[All Fields] AND "products"[All Fields]) OR "biological products"[All Fields] OR "biologic"[All Fields] OR "biologicals"[All Fields] OR "biological factors"[MeSH Terms] OR ("biological"[All Fields] AND "factors"[All Fields]) OR "biological factors"[All Fields] OR "biologics"[All Fields] OR "biologically"[All Fields] OR "biology"[MeSH Terms] OR "biology"[All Fields] OR "biological"[All Fields]) AND "disease-modifying"[All Fields] 9,796
31 "biological therapy"[MeSH Terms] 752,683
32 "biological products"[MeSH Terms] OR ("biological"[All Fields] AND "products"[All Fields]) OR "biological products"[All Fields] OR ("biological"[All Fields] AND "drugs"[All Fields]) OR "biological drugs"[All Fields] 797,661
33 "biological products"[MeSH Terms] 650,188
34 "cdmard"[All Fields] OR "cdmards"[All Fields] 130
35 "interval prolongation"[All Fields] 2,431
36 "reduction strategy"[All Fields] 1,361
37 "adjust*"[All Fields] OR "de-escalation"[All Fields] OR "discontinu*"[All Fields] OR ("dose"[All Fields] AND ("adaptation, psychological"[MeSH Terms] OR ("adaptation"[All Fields] AND "psychological"[All Fields]) OR "psychological adaptation"[All Fields] OR "adjustment"[All Fields] OR "adjust"[All Fields] OR "adjusted"[All Fields] OR "adjusting"[All Fields] OR "adjustments"[All Fields] OR "adjusts"[All Fields])) OR ("drug tapering"[MeSH Terms] OR ("drug"[All Fields] AND "tapering"[All Fields]) OR "drug tapering"[All Fields] OR ("dose"[All Fields] AND "reduction"[All Fields]) OR "dose reduction"[All Fields]) OR ("dose"[All Fields] AND ("spaced"[All Fields] OR "spacing"[All Fields] OR "spacings"[All Fields])) OR ("drug tapering"[MeSH Terms] OR ("drug"[All Fields] AND "tapering"[All Fields]) OR "drug tapering"[All Fields] OR ("dose"[All Fields] AND "tapering"[All Fields]) OR "dose tapering"[All Fields]) OR ("dose-adjustment"[All Fields] AND ("strategie"[All Fields] OR "strategies"[All Fields] OR "strategy"[All Fields] OR "strategy s"[All Fields])) OR (("dosed"[All Fields] OR "doses"[All Fields] OR "dosing"[All Fields] OR "dosings"[All Fields]) AND "down"[All Fields]) OR ("down"[All Fields] AND "titrat*"[All Fields]) OR "drug dosage calculations"[MeSH Terms] OR "drug tapering"[MeSH Terms] OR "escalat*"[All Fields] OR "interrupt*"[All Fields] OR "interval prolongation"[All Fields] OR "interval*"[All Fields] OR "optimis*"[All Fields] OR "optimiz*"[All Fields] OR "re escalat*"[All Fields] OR "reduction strategy"[All Fields] OR "restart*"[All Fields] OR ("spare"[All Fields] OR "spared"[All Fields] OR "spares"[All Fields] OR "sparing"[All Fields]) OR "stop*"[All Fields] OR "taper*"[All Fields] OR ("taper"[All Fields] OR "tapered"[All Fields] OR "tapering"[All Fields] OR "tapers"[All Fields]) OR (("therapeutics"[MeSH Terms] OR "therapeutics"[All Fields] OR "treatments"[All Fields] OR "therapy"[MeSH Subheading] OR "therapy"[All Fields] OR "treatment"[All Fields] OR "treatment s"[All Fields]) AND ("withdraw"[All Fields] OR "withdrawal"[All Fields] OR "withdrawals"[All Fields] OR "withdrawing"[All Fields] OR "withdraws"[All Fields])) OR "withdraw*"[All Fields] OR "withhold*"[All Fields] 2,760,715
38 "bdmard"[All Fields] OR "bdmards"[All Fields] OR (("biological products"[MeSH Terms] OR ("biological"[All Fields] AND "products"[All Fields]) OR "biological products"[All Fields] OR "biologic"[All Fields] OR "biologicals"[All Fields] OR "biological factors"[MeSH Terms] OR ("biological"[All Fields] AND "factors"[All Fields]) OR "biological factors"[All Fields] OR "biologics"[All Fields] OR "biologically"[All Fields] OR "biology"[MeSH Terms] OR "biology"[All Fields] OR "biological"[All Fields]) AND "disease-modifying"[All Fields]) OR "biological therapy"[MeSH Terms] OR ("biological products"[MeSH Terms] OR ("biological"[All Fields] AND "products"[All Fields]) OR "biological products"[All Fields] OR ("biological"[All Fields] AND "drugs"[All Fields]) OR "biological drugs"[All Fields]) OR "biological products"[MeSH Terms] OR ("cdmard"[All Fields] OR "cdmards"[All Fields]) 1,432,392
39 Search #1 AND #37 and #38 744
40 Search #39 Filters from 2015 - 2022 584

**Database, Platform: Embase**

**Date of search: May 2022**

**Search strategy (results)**

#1,"'psoriasis'/mj NOT ('psoriatic arthritis'/mj OR 'arthritic psoriasis' OR 'arthritis psoriatica' OR 'arthritis, psoriasis' OR 'arthritis, psoriatic' OR 'arthropathic psoriasis' OR 'arthropathy, psoriatic' OR 'psoriasis arthropathica' OR 'psoriasis, arthritis' OR 'psoriatic arthritis' OR 'psoriatic arthropathy' OR 'psoriatic polyarthritis' OR 'psoriatic rheumatism')",38585#9,"#1 AND #7 AND #8",92
#2,"'drug dose reduction'/mj OR 'dosage decrease' OR 'dosage reduction' OR 'dose decrease' OR 'dose reduction' OR 'drug dose reduction' OR 'drug tapering' OR 'reduction of drug dosage' OR 'reduction of drug dose' OR 'reduction, drug dose'",106593#8,"#2 OR #3 OR #4 OR #5 OR #6",133859
#3,"'de escalation'",5674

#4,"'dose adjustment'/exp",22

#5,"'optimization'/exp",56
#6,"'retreatment'/exp OR 'retreatment'",22468

#7,"'biological product'/exp OR 'biologic' OR 'biologic agent' OR 'biologic agents' OR 'biologic product' OR 'biologic products' OR 'biological' OR 'biological agent' OR 'biological agents' OR 'biological product' OR 'biological products' OR 'biologicals' OR 'biologics'",3514990

**Database, Platform: Ovid**

**Date of search: May 2022**

**Search strategy (results)**

1. psoriasis.ab,kw,ti., 9200
2. 'psoriatic arthritis'.ab,kw,ti., 2767
3. (psoriasis not 'psoriatic arthritis').ab,kw,ti., 7805
4. ('dosage decrease' or 'dosage reduction' or 'dose decrease' or 'dose reduction' or 'drug dose reduction' or 'drug tapering').ab,kw,ti., 6192
5. de escalation.ab,kw,ti., 688
6. dose adjustment.ab,kw,ti., 1919
7. optimization.ab,kw,ti., 5752
8. retreatment.ab,kw,ti., 2559
9. ('biological product' or 'biologic' or 'biologic agent' or 'biologic agents' or 'biological' or 'biological agent' or 'biologicals').ab,kw,ti., 29563
10. 4 or 5 or 6 or 7 or 8, 16824
11. 3 and 9 and 10, 18

# **Flowchart of the search, screening and selection of evidence (PRISMA) from databases**

| Identification |  | References identified by searching databases | |  |  | References identified by manual search | |
| --- | --- | --- | --- | --- | --- | --- | --- |
|  |  | n= 694 | |  |  | n=28 | |
|  |  |  |  |  |  |  |  |
| Screening |  | References after removing duplicates | |  |  |  |  |
|  |  | n=696 | |  |  |  |  |
|  |  |  |  |  |  |  |  |
|  |  | Screened references (title/abstract) | |  |  | References excluded  n=616 | |
|  |  | n=696 | |  |  |  |  |
|  |  |  |  |  |  |  |  |
| Eligibility |  | References analyzed in full text  n=80 | |  |  | References excluded  n=31  Arthropathies (PAs)=11  Update included=2  No additional information=5  No intervention=5  Not available full text=6  No study type=2 | |
|  |  |  | |  |  |  |  |
|  |  |  |  |  |  |  |  |
| Inclusion |  | Included studies | |  |  |  |  |
|  |  | n=49 | |  |  |  |  |
|  |  |  |  |  |  |  |  |
|  |  | Clinical trials= 15(18 publications)  Observational studies=22  Reviews/guidelines/consensus=10  Economic studies=2 | |  |  |  |  |

# **Quality assessment of selected studies**

***Clinical trials. Risk of Cochrane bias***

| Bias | Randomization sequence generation (selection bias) | Allocation concealment (selection bias) | Blinding of participants and personnel (performance bias) | Blinding of outcome assessment (detection bias) | Incomplete outcome data (attrition bias) | Selective reporting (reporting bias) | Other sources of bias | Summary of the risk of bias |
| --- | --- | --- | --- | --- | --- | --- | --- | --- |
| Atalay S 2021 | Low risk | Low risk | High risk | High risk | Low risk | Low risk | High risk | High risk |
| Blauvelt A 2017 | Unclear | High risk | Unclear | Unclear | Low risk | Low risk | Low risk | High risk |
| Kimball AB 2020 | Low risk | Unclear | High risk | Low risk | Low risk | Low risk | High risk | High risk |
| Lebwohl M 2015 | Low risk | Unclear | High risk | Low risk | Low risk | Low risk | Low risk | Low risk |
| Reich K 2019 | Low risk | Unclear | High risk | Low risk | Low risk | Low risk | Low risk | Low risk |
| Papp 2011 | High risk | Unclear | High risk | Unclear | Low risk | Low risk | Low risk | High risk |
| Larian A 2011 | Unclear | Unclear | High risk | High risk | Low risk | Low risk | High risk | High risk |
| Papp 2005 | Unclear | Low risk | Low risk | Low risk | Low risk | Low risk | Unclear | Some considerations |
| Gordon 2015 | Unclear | Unclear | High risk | High risk | Low risk | Low risk | Low risk | High risk |
| Ortonne J 2009 | Unclear | Unclear | High risk | High risk | Low risk | Low risk | Low risk | High risk |
| Gordon K 2006 | Low risk | Unclear | Low risk | Low risk | Low risk | Low risk | Unclear | Some considerations |

***Observational studies. Newcastle-Ottawa Scale (NOS)***

| Author  Year of publication | van Bezooijen 2017 | Atalay  2021 | Bagel  2021 | Fotiadou C  2012 | Taniguchi T  2013 | Esposito M  2017 |
| --- | --- | --- | --- | --- | --- | --- |
| Representativeness of the exposed cohort | ⋆ | ⋆ | ⋆ | ⋆ | ⋆ | ⋆ |
| Selection of the unexposed cohort |  |  |  |  |  |  |
| Determination of exposure | ⋆ | ⋆ | ⋆ | ⋆ | ⋆ | ⋆ |
| Demonstration that the cohort was free of the event at the start of the study | ⋆ | ⋆ | ⋆ | ⋆ | ⋆ | ⋆ |
| Comparison of cohorts at the basis of design or analysis |  |  |  |  |  |  |
| Outcome assessment | ⋆ | ⋆ | ⋆ | ⋆ | ⋆ | ⋆ |
| Sufficient length of follow-up for outcomes | ⋆ | ⋆ | ⋆ | ⋆ | ⋆ | ⋆ |
| Adequate cohort follow-up | ⋆ | ⋆ | ⋆ | Treatment time was not the same for everyone | ⋆ | ⋆ |
| Score | 6 | 6 | 6 | 5 | 6 | 6 |
| Design | Follow-up cohort | Retrospective follow-up cohort without hypothesis testing | Retrospective follow-up cohort | Follow-up cohort | Prospective follow-up cohort | Retrospective follow-up cohort without hypothesis testing |

| Author  Year of publication | Hansel k  2017 | Llamas-Velasco M  2020 | López-Ferrer A  2013 | Piaserico S  2016 | Romero-Jimenez  2018 | Cassano N  2010 |
| --- | --- | --- | --- | --- | --- | --- |
| Representativeness of the exposed cohort | ⋆ | ⋆ | ⋆ | * | ⋆ | ⋆ |
| Selection of the unexposed cohort | ⋆ | ⋆ |  | ETA compared to ADA | ⋆ |  |
| Determination of exposure | ⋆ | ⋆ | ⋆ | ⋆ | ⋆ | ⋆ |
| Demonstration that the cohort was free of the event at the start of the study | ⋆ | ⋆ | ⋆ | ⋆ | ⋆ | ⋆ |
| Comparison of cohorts at the basis of design or analysis | ⋆ | ⋆ |  | ⋆ | ⋆ |  |
| Outcome assessment | ⋆ | ⋆ | ⋆ | Unclear | ⋆ | ⋆ |
| Sufficient length of follow-up for outcomes | ⋆ | ⋆ | Follow-up was short; participants did not receive treatment for the same length of time. | ⋆ | ⋆ | ⋆ |
| Adequate cohort follow-up | ⋆ | ⋆ | ⋆ | No es claro | ⋆ | ⋆ |
| Score | 8 | 8 | 5 | 5 | 8 | 6 |
| Design | Cohort study | Cohort study | Follow-up cohort | Retrospective cohort | Cohort study | Follow-up cohort |

***Cross-sectional studies. Checklist for Analytical Cross-Sectional Studies, JBI***

| Author, Year of publication | Baniandrés O,  2015 | Carrascosa JM,  2015 | Romero-Jiménez,  2016 |
| --- | --- | --- | --- |
| 1. Were the criteria for inclusion in the sample clearly defined | Yes | Yes | Yes |
| 2. Were the study subject and the setting described in detail | Yes | Yes | Yes |
| 3. Was the exposure measure in valid and reliable way | Yes | Yes | Yes |
| 4. Were objective, standard criteria used for measurement of the condition? | Yes | Yes | Yes |
| 5. Were confounding factors identified | Yes | Yes | Yes |
| 6. Were strategies to deal with confounding factor stated | Yes | Yes | Yes |
| 7. Was the outcome measured in a valid and reliable way | Yes | Yes | Yes |
| 8. Was appropriate statistical analysis used | Yes | Yes | Yes |

**Supplementary material 2. Delphi consensus process**

For the consensus rounds, the questionnaire constructed from the information extracted from the evidence was shared with the experts. The assessment of the statements was made using a Likert-type scale, as indicated by the "appropriate use method" developed by the *RAND Corporation* and the University of California at Los Angeles (UCLA).^[[1]](#footnote-1)^ . The response categories are located in three areas (1-2 disagreement; 3 neither agreement nor disagreement; 4-5 agreement). Each panelist scored each statement according to the degree of agreement with the statement.

The first round of responses to the questionnaire was carried out "blindly" to the judgment of the other members of the panel through a virtual questionnaire. The analysis of the information of the round was carried out by means of frequency distribution and measures of central tendency, classifying each item in consensus scenarios according to the level of agreement and the direction of the recommendation, as presented in the following table:

Analysis parameters for the consensus voting method Delphi

| **Agreement level**  **Consensus: If the extreme points of the range of responses are located within any of the three regions of the scale zone (1-2; 3; 4-5).**  **If the extreme points of the range are located within two consecutive regions or <30% of panelists scored outside the region that contains the median.**  **No consensus** : The scores of a minimum of 30% of panelists are in the region of 1-2, and a minimum of 30% in the region of 4-5 or the interquartile range ≥ 3.  **Indeterminate** : Statements in which there is neither agreement nor disagreement. |
| --- |
| **Direction of recommendation**  **Agree** : median in interval 4-5 and without disagreement.  **Disagreement** : median in interval 1-2 and without disagreement.  **Doubtful** : median in 3 or no consensus. |

A second round was carried out synchronously anonymously in a debate session where new evaluations were carried out (round 2) and recommendations were reformulated. All experts participated in the two decision rounds.

**Supplementary material 3. Characteristics of the included studies**

**Selected clinical trials on dose reduction or extension of biological intervals in patients with psoriasis**

| Author, year | Atalay S, 2020 & 2021 | Blauvelt et al., 2017 | Lebwohl , 2015 | Reich et al., 2020 | Papp 2005 | Gordon2007 & Larian 2011 | Moore 2007 | Schäkel 2022 GUIDE study (abstract) |
| --- | --- | --- | --- | --- | --- | --- | --- | --- |
| Study design | Clinical trial, randomized, multicenter, open-label. CONDOR | Clinical trial, randomized, controlled, double-blind, PSTELLAR | Clinical trial, randomized, controlled, double-blind, phase III. AMAGINE‐2, AMAGINE‐3 | Clinical trial, randomize, controlled, open-label, blinded assessor, phase IIIB | Clinical trial, randomized, controlled, double-blind, phase 3 | Clinical trial, randomized, multicenter, controlled, double-blind, open-label | Clinical trial, randomized, controlled, open-label | Clinical trial, randomized, controlled, double-blind, phase IIIB, parallel groups |
| Place | Netherlands, 6 centers | USA, Canada and Belgium, 48 centers | Multicenter, 142 centers around the world | Europa, 202 centers | United States, Canada and Western Europe | United States, 16 centers | United states | Multicentric |
| Population | Patients with psoriasis with low activity. | Adults (18–80 years) with moderate to severe plaque psoriasis. | Patients aged 18 to 75 years with moderate to severe plaque psoriasis. | Moderate to severe psoriasis. | Patients over 18 years of age with stable psoriasis. | Patients with moderate-severe plaque psoriasis from Study M02-538 | > 18 years or older, with stable and active plaque psoriasis with BSA≥ 10%. | Psoriasis patients |
| N | 111 (53 de-escalation, 58 standard doses) | 378 (196 de-escalation, 84 failed de-escalation, 76 standard doses and 22 lost to follow-up) | 2374 (1698 de-escalation, 676 standard doses) | 1306 (662 de-escalation, 644 standard dose) | 583 in total, 194 in de-escalation | 148 | 2546 (1274 de-escalation) | 822 in part 2 of the study, 297 (36.1%) superresponders* (n=148 GUS every 8 weeks and n=149 GUS every 16 weeks), and 525 (63.9%) non-superresponders |
| Biological * | ADA, ETN, UST | UST | BRO | SEC | ETN | ADA | ETN 50 mg twice a week | GUS every 8 weeks |
| Duration of treatment prior to de-escalation (weeks) | ≥24 | 28 | 12 | ≥24 | No previous ETN or anti-TNF | 24 | 12 | 20-28 |
| Eligibility criteria for de-escalation | PASI and DLQI ≤5 baseline, PASI ≤5 for ≥6 months before baseline | PGA 0/1 a week 28 | PASI 12 or more, sPGA 3 or more, and involvement of 10% or more of the body surface area | ≥ PASI90 | PASI ≤ 10 at screening; and have received or be a candidate for systemic therapy or phototherapy. | PASI ≥50 | PGA≤2 and improvement from baseline | Absolute PASI = 0 at week 20 and at week 28 with GUS 100 mg every 8 weeks. |
| De-escalation strategy | Stepped extension to:  ADA every 3 or 4 weeks  ETN every 10 days or 2 weeks  UST every 18 or 24 weeks  Second prolongation after 3 months if PASI and DLQI ≤ 5 | Based on the dose interval determination period: UST every 12, 16, 20 or 24 weeks | Fixed interval:  140 mg every 2 weeks  (N=680) o 140 mg every 4 weeks (N=676) or 140 mg every 8 weeks (N=342) or 210 mg every 2 weeks (N= 676) | Fixed interval: SEC 300 mg every 6 weeks | Randomized to receive for 12 weeks: ETN 50 mg twice a week, ETN 25 mg twice a week, or placebo twice a week. Week 13 to 24: all ETN 25 mg twice a week. | At week 12, patients with weekly ADA 40 who presented PASI ≥50 were randomized blind to ADA 40 mg every 2 weeks or placebo for a further 12 weeks. | Weeks 12-24: Continuous treatment of ETN at reduced dose 50 mg once a week (n = 1272) or discontinuation (n = 1274 until relapse (loss of PGA≤2) | Superresponders: GUS 100 mg every 8 weeks (weeks 28, 36, 44, 52, 60) or every 16 weeks  Nonsuperresponders: GUS 100 mg every 8 weeks |
| Monitoring during de-escalation | Every 3 months  Total: 12 months | Total 124 weeks: open period of 28 weeks, treatment and double-blind follow-up of 96 weeks. | 40 weeks (after 12 weeks of induction phase) | 28 weeks (after 24 weeks of induction phase) | Improvement ≤75% in PASI (PASI 75) at 12 weeks. Total: 24 weeks | Initial 12, 24  12, 24 in retreatment. Total: 48 weeks | 12, 16, 20, 24 | Every 8 weeks for 40 weeks after de-escalation |
| Treatment results | Dose reduced successfully:  N = 28 (53%) (N = 16 ADA, N = 3 ETN, N = 9 UST). N = 10 took 67% of the original dose and N = 18 took 50% of the original dose.  De-escalation group vs. standard dose group:  Median PASI: 3.4 vs. 2.1. Median DLQI: 1.0 vs. 0. Short shoots: 36% vs. 14%. Persistent outbreak: 9% vs. 9% | 70% (N = 196) of patients in de-escalation and 70% (N = 53%) of patients with standard doses maintained a PASI75 response in the seven visits of the evaluation period. | Maintenance of sPGA 0/1 to week 52 - De-escalation vs. standard dose:  AMAGINE-2: 140 mg every 2 weeks: N=144 (43%); 95% CI 37-48); 140 mg every 4 weeks: N=30 ((9%); 95% CI 6-13); 140 mg every 8 weeks: N=8 ((5%); 95% CI 2-9) vs. 210 mg every 2 weeks: N=209 ((63%), 95% CI 57-68).  AMAGINE-3: 140 mg every 2 weeks: N=154 ((45%); 95% CI 40-50); 140 mg every 4 weeks: N=53 ((16%); 95% CI 12-20); 140 mg every 8 weeks: N=10 ((6%); 95% CI 3-10) vs. 210 mg every 2 weeks: N=208 ((61%); 95% CI 55-66).  In both trials, 210 mg every 2 weeks showed a significant difference in effect compared to 140 mg every 2/4/8 week. | At week 52, 85.7% of patients in the standard dose group (every 4 weeks) vs. 74.9% of de-escalation patients (every 6 weeks) maintained the PASI90 response (OR 1.91; 95% CI: 1.44-2.55). Both groups (every 4 weeks vs. every 6 weeks) did not differ markedly in their PASI50 (99.7% vs. 99.2%) and PASI75 (97.9% vs. 93.5%) responses at week 52. | 3 patients suspended due to: Adverse event (1), Patient rejection (1), Loss of follow-up (1). At 12 weeks after dose reduction, 54% of the patients achieved PASI 75. 32% of the 88 patients who had not achieved a PASI 75 response at week 12 (in all groups) did so at week 24, a despite the decrease in the dose of ETN from 50 mg twice a week to 25 mg twice a week | Additionally, 48.5% (33/68) of patients assigned to placebo at week 12 responded to PASI 75 at week 24, compared with 67.6% (46/68) of patients assigned to ADA 40 mg every 2 weeks (p = 0.032). Additionally, 27.9% (19/68) of patients assigned to placebo at week 12 responded to PASI 90 at week 24, compared with 47.1% (32/68) of patients assigned to ADA 40 mg every 2 weeks | Maintenance rate of response at week 24:  Continuous: 84.5%  Discontinued: 68.9%  (p <0.0001) | 92.6% and 91.9% of GUS patients every 8 or 16 weeks, respectively, achieved PASI <3 at 68 months (P = 0.001). Of the SRs with GUS every 16 weeks, 79.2% maintained PASI ≤1 and 69.1% PASI = 0 at week 68. The SRs also achieved and maintained a high DLQI response rate of 0/1 until week 68, regardless of the dosing interval. |
| Definition of failure | PASI >5 y/o DLQI >5 | - | Single sPGA score ≥3 or persistent sPGA scores of 2 for ≥4 weeks to week 16 | - | < PASI75 a week 12 | Improvement of <PASI 50 within 12 weeks following reduction in dosing frequency or discontinuation of treatment. | Loss of response status to the PGA.  Respondent: PGA≤ 2 and improvement over baseline. | No maintenance of PASI <3, DLQI <5 |
| Time to relapse | The first persistent outbreak occurred after 7.5 months in the reduction group vs. 3 months in the standard dose group | - | No data | - | 2 patients discontinued due to lack of efficacy. | Of those randomized to placebo at week 12, 30.9% (21/68) experienced relapse at week 24, compared with 16.2% (11/68) of those who received ADA 40 mg every 2 weeks. | Mean (median) time to relapse: 39.6(33.0) days | - |
| Retreatment strategy | Retreatment with the previous effective dose. | - | BRO 210 mg every 2 weeks | - | No data | New open treatment with 12 weeks of weekly ADA. | After a relapse at week 16 or 20, ETN 50 mg was restarted once a week until week 24. | - |
| Retreatment results | At 12 months, N = 4 in the de-escalation group versus N = 1 in the standard dose group with a persistent flare had not recovered PASI and DLQI ≤5 | - | No data | - | No data | 81.3% (26/32) returned to PASI50 after 12 weeks of retreatment. Of the 19 M03-596 patients with PASI 75 at week 12 of M02-538, 12 (63.1%) returned to achieve PASI 75 at week 12 of M03-596. | Response time to recovery: 35.0 (29.0) days | - |

* PASI 0 at 12 weeks

ADA adalimumab (standard dose of 40 mg every 2 weeks), ETN etanercept (standard dose of 50 mg once weekly), GUS guselkumab, IFX infliximab (standard dose of 5 mg/kg every 8 weeks), UST ustekinumab (standard dose of 45 mg every 12 weeks (≤100 kg) or 90 mg every 12 weeks (> 100 kg)), SEC secukinumab (standard dose of 300 mg every 4 weeks), and BRO brodalumab (standard dose of 210 mg every 2 weeks) were used.

DLQI Quality of Life Index in Dermatology; PGA Physician's Global Assessment; PASI Area and Severity Index of Psoriasis; PASI50/75/90/100 50/75/90/100% Improvement in the Area and Severity Index of Psoriasis; sPGA = Static Physician's Global Assessment; 95% CI = 95% confidence interval; SD = standard deviation.

**Selected clinical trials involving complete suspensions of biological material from patients with psoriasis**

| Author, year | Gordon 2006 | Gordon 2014 | Kimball AB, 2020 | Ortonne 2008 & Ortonne 2009 | Papp 2011 | Menter 2008 | Blauvelt 2020 |
| --- | --- | --- | --- | --- | --- | --- | --- |
| Study design | Randomized, controlled, double-blind clinical trial | Post hoc analysis of open clinical trial (*NCT00195676*) | Randomized clinical trial, reSURFACE 1 and reSURFACE 2 | Post hoc analysis of the clinical trial, open (CRYSTEL) | Open Clinical Trial Extension (NCT  00195676) | Randomized, controlled, double-blind clinical trial, phase 3 | Clinical trial, phase 3, randomized, double-blind, placebo-controlled |
| Place | United States, 47 centers | United States, Canada and Europe, 104 centers | reSURFACE 1 (118 centers in Australia, Canada, Japan, UK and USA) and reSURFACE 2 (132 centers in Europe, Israel and the United States) | Europe and Asia, 119 centers in 19 countries | Canada, Europe and the United States | 67 centers in the United States and 14 centers in Canada | Multinational |
| Population | Patients between 18 and 84 years old, with active but clinically stable plaque psoriasis,> 10% of the body surface, PASI10 during the screening period. | Patients ≥ 18 years of age, with a clinical diagnosis of psoriasis for ≥6 months, BSA> 10% in REVEAL and CHAMPION and ≥5% in M02-528, and PASI ≥10 in CHAMPION and ≥12 in REVEAL. | Adults with moderate to severe chronic plaque psoriasis. | Moderate to severe psoriasis patients included from a large multicenter trial. | Patients with moderate to severe psoriasis. | Patients ≥ 18 years of age, with a clinical diagnosis of moderate to severe psoriasis, for ≥6 months | Patients with moderate to severe chronic plaque psoriasis stable for ≥6 months, BSA ≥10%, PASI ≥12 and sPGA ≥3 |
| N | 652 (409 de-escalation) | 133 | reSURFACE 1: 676  reSURFACE 2: 794 | 363 | 347 | 1212, 240 on drug recall | 336 |
| Biological * | ETN 50 mg twice a week, 25 mg twice a week, or 25 mg once a week | ADA | tildrakizumab 100 mg and 200 mg | ETN 2 times a week 50 mg | ADA 40 mg every 2 weeks or once a week | ADA 40 mg every 2 weeks | RIS q12 week |
| Duration of treatment prior to de-escalation (weeks) | At least one previous treatment of phototherapy or systemic therapy for psoriasis, or candidate for these. | 16 weeks | 28 weeks | 12 weeks | 104-252 week | 16 | 28 |
| Eligibility criteria for de-escalation | ≥ PASI50 a week 24 | PASI 75 at week 16, PGA of doctor 0 or 1 | PASI 75 at week 28 | PGA≤2 | PGA≤2 | ≥PASI75 at week 16 and week 33 of the study were reassigned to treatment withdrawal (n = 240) or to continuous treatment with 40 mg every 2 weeks (n = 250) | sPGA 0/1 |
| De-escalation strategy | Complete suspension of ETN until relapse of the disease. | After ADA 40 mg every 2 weeks, complete withdrawal of treatment (up to 40 weeks). | Complete discontinuation of medication | Complete suspension of treatment. | Complete suspension of treatment until relapse or 40 weeks. | Complete suspension of treatment. | Week 28: 111 subjects to RIS every 12 weeks and 225 withdrawals from treatment |
| Monitoring during de-escalation | weeks 26, 28, and every 4 weeks up to 48 weeks | 16 weeks retreatment. Total: 40 weeks | Semanas 32,40,52 (reSURFACE2) y 64 (reSURFACE1) | Every 3 months. Total: 54 weeks | Every 4 weeks. Total: 40 weeks | ADA withdrawal at week 33. Follow-up at weeks 36, 40, 44, 48 and 52. | From week 28, every 12 weeks until week 104 |
| Treatment results | For the subset of patients who achieved PASI 75 at week 24 (n = 252):  (1) Median time to loss of PASI 50: 91 days  (Bottom 25% of patients 58 days, top 25% of patients 170 days)  (2) Median time to loss of PASI 75: 57 days | During drug withdrawal, HRQL worsened disproportionately compared to signs of skin disease; HRQL also worsened considerably in patients who did not relapse.  The patients recovered their HRQL after retreatment with ADA. | The results were similar for those who had achieved PASI 90, PASI 100 and improvement in PGA.  reSURFACE1: 49% of patients who had reached PASI75 at week 28 did not develop relapses at 64 weeks after stopping.  reSURFACE2: 56.7% of patients who had reached PASI75 at week 28 did not develop relapses at 64 weeks after stopping. | The mean PGA was significantly reduced from the start (3,6). The mean PASI decreased significantly from baseline (9.5). PASI improved 59% from baseline to week 54 in patients who discontinued ETN.  Median response time (PGA≤2) to ETN in initial treatment: 11 weeks  Unsatisfactory efficacy response (n = 67; 18.5%)  Patient request (n = 23; 6.3%)  Adverse event (n = 13; 3.6%) | 178/285 relapsed before restarting treatment and 107/285 did not relapse. | Percentage of pts. who "lose an adequate response" (PASI <50 and a 6-point increase in PASI relative to the 33rd week score) after 33rd week and on or before week 52:  28% (68/240) of those who discontinued treatment and 5% (12/250) of those who had continuous treatment with 40 mg every 2 weeks (p <0.001).  In the drug withdrawal arm, 5 patients discontinued due to unsatisfactory effect. | At week 52, 97 patients (87.4%) who received RIS reached sPGA 0/1 vs. 138 patients (61.3%) on placebo. At week 104, 90 patients (81.1%) who received risankizumab reached sPGA 0/1 compared to 16 patients (7.1%) who received placebo (differences adjusted for placebo: week 52: 25.9%; CI 95%, 17.3% -34.6%; week 104: 73.9%; 95% CI, 66.0% -81.9%; p <0.001 for both). Treatment-emergent adverse event rates were similar between risankizumab (186 [45.7%]) and placebo (49 [49.0%]) in Part A1 and were stable over time. |
| Definition of failure | Loss of a PASI improvement> 50% at week 24 | Loss of ≥50% of initial improvement in PASI | 50% reduction in maximum PASI response after treatment withdrawal | Relapse was based on the opinion of the investigator but should be considered as "moderate" psoriasis or worse (PGA ≥3). | PGA ≥3 | - | sPGA ≥3 after week 32 |
| Time to relapse | Median time to relapse: 85 days | 24/133 (18%) relapsed during treatment withdrawal. | Average 24 weeks, both groups  reSURFACE1: 0.9% (week 32), 5.5% (week 40), 10.8% (week 52) and 11.8% (week 64).  reSURFACE2: 0.8% (week 32), 3.5% (week 40), 14.1% (week 52) and 10.0% (week 64) | relapse of PGA> 3 | relapse of PGA≥3. Median time to relapse = 141 days (interquartile range 93-202 days) | - | At 4 weeks of de-escalation, 4/111 had failure and 153/225 of those were on placebo (withdrawal of RIS) |
| Retreatment strategy | Blind ETN with the same dose that they received in week 24 of the double-blind period, followed every 4 weeks and portrayed for up to 24 weeks. | All patients were treated again with an initial dose of ADA of 80 mg, followed by 40 mg every 2 weeks for 16 weeks. | Resume assigned dose at randomization.   reSURFACE1: 100 mg  reSURFACE2: 200 mg | The patients were treated again with 25 mg of ETN twice a week until week 54 of the study. | Retreatment after relapse (n = 178) or when they reached a maximum of 40 weeks of abstinence (n = 107) with 80 mg at week 0 followed by 40 mg every 2 weeks for 16 weeks | - | Risankizumab 150 mg |
| Retreatment results | Differences in PASI between week 12 of initial treatment and week 12 of retreatment (mean 5.8 versus mean 6.4): Difference in means per patient: -0.05 (95% CI: -1.1 to 0)  Efficacy of retreatment (n = 297): PASI responders at week 12 of initial treatment who achieved PASI 50 or 75 at week 12 of retreatment  PASI 50 to PASI 50: 83%.  PASI 75 to PASI 50: 93%.  PASI 75 to PASI 75: 52%. | After 16 weeks of retreatment, 75% of those who relapsed and 89.9% of those who did not relapse had a PASI 75 response. | reSURFACE1: 86% achieved PASI 75 at 64 weeks   reSURFACE2: 83% achieved PASI 75 at 64 weeks. | Recovery of PGA≤2 after retreatment 187/226 (83%). Mean response time (PGA≤2) to ETN in retreatment: 15 weeks (p = 0.001 versus initial response) | Overall PGA rate 0 ⁄1 after 16 weeks of retreatment: 76%. For those who relapsed during the withdrawal period: 69% (123/178)  For those who did not relapse in the withdrawal period: 89% (95/107) | - | - |

ADA adalimumab (standard dose of 40 mg every 2 weeks), ETN etanercept (standard dose of 50 mg once weekly), GUS guselkumab, IFX infliximab (standard dose of 5 mg/kg every 8 weeks), UST ustekinumab (standard dose of 45 mg every 12 weeks (≤100 kg) or 90 mg every 12 weeks (> 100 kg)), SEC secukinumab (standard dose of 300 mg every 4 weeks), and BRO brodalumab (standard dose of 210 mg every 2 weeks) were used.

DLQI Quality of Life Index in Dermatology; PGA Physician's Global Assessment; PASI Area and Severity Index of Psoriasis; PASI50/75/90/100 50/75/90/100% Improvement in the Area and Severity Index of Psoriasis; sPGA = Static Physician's Global Assessment; 95% CI = 95% confidence interval; SD = standard deviation

**Selected observational studies on biological de-escalation in patients with psoriasis**

| Author, year | Atalay S, 2021 | Baniandrés 2015 | Bardazzi et al., 2016 | Carrascosa 2015 | Esposito M 2017 |
| --- | --- | --- | --- | --- | --- |
| Study design | Observational, prospective (Not randomized, controlled) | Observational, retrospective cohort (uncontrolled) | Observational, retrospective cohort (uncontrolled) | Observational, retrospective cohort (uncontrolled). BIOBADADER  M | Observational, retrospective cohort (uncontrolled). Wave |
| Place | Netherlands, 1 center | Madrid, Spain | Italy, 2 centers | BIOBADADERM registry, 9 centers | Italy, 3 centers |
| Population | Psoriasis patients with low disease activity. | Patients with moderate-severe psoriasis. | Patients with moderate to severe psoriasis. | Patients with severe psoriasis | Psoriasis patients |
| N | 498 eligible (80 included in the analysis) | 104 (56 de-escalation, 48 standard doses) | 20 (all in de-escalation) | 637 (223 de-escalation, 368 standard doses) | 350 (47 de-escalation of which 27 due to persistent remission)  g |
| Biological * | ADA, ETN, UST | ADA, ETN, IFX, UST | IFX | ADA, ETN, IFX, UST | ADA, ETN, IFX (UST without de-escalation) |
| Duration of treatment prior to de-escalation (weeks) | ≥6 meses | ≥ 6 months | ≥ 12 meses | Average 149.6 (SD 78.6) week | "Prolonged remission" |
| Eligibility criteria for de-escalation | PASI and DLQI ≤5 at baseline, with PASI ≤5 for ≥6 months before baseline, or those authorized by your treating physician | PASI90 ≥ 6 meses | PASI 0 ≥ 12 months |  | "Remission" |
| De-escalation strategy | Staggered extension, started conservatively with a single step. The interval was prolonged, reaching 67% of the original dose. | Stepped extension to:  ADA every 3, 4 or 6 weeks  ETN 25 mg once a week or 50 mg every 2 weeks  IFX every 9 or 11 weeks  UST 45 mg every 13 or 14 weeks | Fixed interval: 5 mg/kg every 10 weeks | Dose adjustment: ADA, ETN, UST and IFX (not specified) | Dose adjustment: ADA, ETN, IFX (unspecified) |
| Monitoring during de-escalation | 670 days (First analysis at 6 months) (Second analysis at 1 year). Evaluation every 3 months. | Not specified | Average 83 ± 22 months of treatment | Evaluation in periods of 3 months | 48 y 96 week |
| Treatment results | 36 discontinued de-escalation: 19 ADA, 7 ETN, 10 UST  7/36 discontinued de-escalation due to PASI or DLQI >5  22/36 discontinued de-escalation despite PASI or DLQI <5  7/36 discontinued de-escalation with PASI and unknown DLQI.  22.7% reduction in dose and costs after 1st year.  44 (55%) patients continued de-escalation at 1-year  PASI: median [IQR], 1.6 [0.2–2.5] at baseline, 1.7 [0.5–3.0] at month 6, and 1.9 [0.8–2.8] at month 12.  DLQI: median [IQR] 0 [0–1] at baseline, 0 [0–1.5] at month 6 and 0.5 [0–2] at month 12.  Skindex-29: median [IQR] 5.2 [0.9–16.4] at baseline, 5.2 [0.9–13.8] at month 6, and 6.9 [0.9–14.7] at month 12. | All patients in de-escalation achieved and maintained ≥PASI75 | N = 5 (25%) of patients in de-escalation experienced a relapse. | De-escalation versus standard dose: mean PASI of 1 (N = 140) versus mean PASI of 2.6 (N = 231) at the cutoff date. 5% improvement in PASI 1.08 (1.01-1.15) | The PASI75 response rate at week 96 was higher in the de-escalation group compared to the standard dose group (not significant). |
| Definition of failure | PASI and/or DLQI> 5 | Loss of response PASI90-100 | Loss of PASI50 response | - | No data |
| Time to relapse | 19 months (95% CI 14.9–23.1) (Time after abandonment of de-escalation, some resumed doses increase regardless of PASI or DLQI status) | No data | No data | - | No data |
| Retreatment strategy | Retreatment with previous effective dose in case of outbreak or at the request of the patient. | Retreatment with standard doses | Retreatment with standard doses. | - | Return to the last effective dose or the standard regimen.  Use of rescue therapy or biological change. |
| Retreatment results | No data | No data | No data | - | No data |

| Author, year | Fotiadou et al., 2012 | Hansel et al., 2017 | Lee ,2018 | Llamas-Velasco M, 2020 | López‐Ferrer et al.,2013 |
| --- | --- | --- | --- | --- | --- |
| Study design | Observational, retrospective cohort (uncontrolled) | Observational, retrospective cohort (uncontrolled) | Observational, retrospective cohort (uncontrolled) | Observational, longitudinal, retrospective | Observational, retrospective cohort (uncontrolled) |
| Place | Greece, 1 reference center | Italy, 1 center | USA, 1 center | Madrid, Spain | Spain, 1 center |
| Population | Patients with moderate to severe psoriasis. | Long-lasting moderate to severe psoriasis. | Patients with psoriasis without the use of prior biologics for ADA. | Adults with moderate to severe psoriasis. | Moderate to severe psoriasis. |
| N | 52 (14 desescalamiento) | 30 (all in de-escalation) | 62 (22 desescalamiento) | 214 patients (99 de-escalation) | 119 (32 desescalamiento) |
| Biological * | ADA | ADA | ADA | ETN, ADA, UTK | ADA |
| Duration of treatment prior to de-escalation (weeks) | ≥ 12 meses | ≥ 12 meses | 3 months | ≥6 meses | Minimum 16 weeks per protocol. |
| Eligibility criteria for de-escalation | PASI100 after 12 months h | PASI100 sustained for ≥ 12 months | Prolongation due to "well controlled disease" (no ADA for dose reduction) ". | Have achieved PASI 90 | PASI75 response in the first 16 weeks of treatment and then persistent PASI <5 |
| De-escalation strategy | Fixed interval: ADA every 3 weeks | Stepped extension:  With 3-4 days every month until ADA every 3 weeks-every 4 weeks | Dose adjustment: ADA> every 2 weeks, up to every 4 weeks (unspecified) | Increased intervals.  In most patients (78.8%), the longest interval between treatment was between 1.2 and 2.0 years. | Dose adjustment: ADA every 3 weeks-every 4 weeks (unspecified) for time periods |
| Monitoring during de-escalation | 30 months | Median 60 months (min. 36, max. 66 months). | 6 months | At 1 month, 3-4 months, and every 3 months.  Months 0, 6, 12, 18 and 24 are reported | 12 months |
| Treatment results | All patients in de-escalation maintained the PASI100 response, and 10 patients (71%) completed the 30-month follow-up (from the start of treatment). | 18 patients in de-escalation (60%) maintained PASI100 (3 patients with ADA every 3 weeks and 15 patients with ADA every 4 weeks). | In patients with remission, 8/9 maintained good control for at least 6 months after dose reduction; 1/9 was lost to follow up | 88.7% of the patients achieved PASI 90 before switching to reduced doses and 24 months later, 61.9% still achieved PASI90 despite the dose reduction.  The “reduced dose” treatment strategy was used in 99 patients (6.1% with ETN, 38.4% with ADA and 55.6% with UTK) | The treatment retention rate was 31/32 (97%) in patients with a long interval. 3/32 (9.4%) of patients with de-escalation reinstated their previous effective dose due to partial loss of response. |
| Definition of failure | No data | PASI response loss ≥20% | - | - | Partial loss of response (unspecified) |
| Time to relapse | No relapse was observed. | The average time to relapse (N=12) was 3.8 months (SD 0.9 months) (median: 3 months (95% CI: 1.9-4.1)) | - | - | No data |
| Retreatment strategy | - | Retreatment with standard doses. | - | - | Retreatment with the previous effective dose. |
| Retreatment results | - | All patients who relapsed (N = 12) achieved a PASI100 ± 1 month after retreatment with the standard dose. | - | - | No data |

| Author, year | Ovejero‐Benito et al., 2020 | Piaserico et al., 2016 | Puig , 2013 | Romero‐Jimenez et al., 2016 | Romero‐Jimenez et al., 2018 |
| --- | --- | --- | --- | --- | --- |
| Study design | Observational, prospective cohort (uncontrolled) | Observational, retrospective cohort (uncontrolled) | Observational, retrospective cohort (uncontrolled) | Observational, retrospective cohort (uncontrolled) | Observational, retrospective cohort (uncontrolled) |
| Place | Madrid, Spain | Italy, 5 centers | Spain | Spain | Spain |
| Population | Moderate to severe psoriasis. | Psoriasis | Moderate to severe psoriasis. | > 18 years with moderate to severe psoriasis in management with the same biological for more than 6 months | > 18 years with moderate to severe psoriasis. |
| N | 120 (183 treatment cycles, reduction in 59 cycles and standard dose in 124 cycles) | 85 (all in de-escalation) | 78 (9 de-escalation) | 224 (118 in the postprotocolization group, of which 39 in de-escalation) | 62 (14 de-escalation, 34 standards doses) k |
| Biological * | ADA, ETN, UST | ADA, ETN | ADA, ETN, IFX, UST (IFX and UST without de-escalation) | ADA, ETN, IFX, UST | UST |
| Duration of treatment prior to de-escalation (weeks) | 2 consecutive visits (not specified) | ≥ 12 meses | >6 meses | ≥ 6 months | Median (p25;p75) time to de-escalation 22.6 months (10.0;37.0) |
| Eligibility criteria for de-escalation | PASI90 or PASI <3 in 2 consecutive visits | PASI 0 ≥ 12 months | At least 24 weeks of treatment with maintenance response PASI75 and absolute PASI <5 | PASI90–100 ≥ 6 meses | Achieve PASI 75 in week 28 |
| De-escalation strategy | Dose reduction included lengthening of the dosage interval (the vast majority) and/or reduction of the dose of the drug by single administration | Fixed interval:  ADA every 3 weeks  ETN every 10 days | Dose adjustment: ADA, ETN (unspecified) | Stepped extension:  ADA every 3 weeks  ETN every 10 days  IFX every 9 weeks  UST every 13 weeks  The intervals could be extended further in the same way if the remission continues for another 6 months | Dose adjustment: UST (unspecified) |
| Monitoring during de-escalation | 6 months | ≥ 12 meses | 24 weeks | ≤ 12 meses | minimum 28 weeks |
| Treatment results | Successful reduction: 96% (26/27) in ADA, 100% (3/3) in ETN and 76% (22/29) in UST patients (183 drug cycles in total). |  | Based on the inclusion criteria of the study, it was assumed that de-escalation patients should have obtained at least one response ≥ PASI75 and a PASI <5. | The dose was lower in 43.4% of ADA patients (N = 23), in 37.5% of ETN patients (N = 9), in 28.6% of INF patients (N = 2) and in 14.7% of UST patients (N = 5). | 6 patients temporarily suspended the biological. It does not mention cause, outcomes, or follow-up. |
| Definition of failure | De-escalation was considered successful if PASI90 was maintained or <3 was maintained for ≥ 6 months. | ≥ 50% loss of PASI improvement | - | Loss of PASI90-100 | - |
| Time to relapse | NA | Average time to relapse 48 months (95% CI 43-52.7) for ADA and 39.3 months (95% CI 33.7-44.8) for ETN.  Cumulative risk of relapse: 0%, 12% and 20% for ADA and 14%, 31% and 39% for ETN after 3, 6 and 12 months, respectively. | - | No data | - |
| Retreatment strategy | NA | Retreatment with the standard dose. | - | Retreatment with the effective dose. | - |
| Retreatment results | NA | All patients who experienced a relapse and were treated with the standard dose showed a rapid response (unspecified) | - |  | - |

| Author, year | Taniguchi 2013 | van Bezooijen 2017 | Cassano 2010 | Castro-Ayarza 2022 | Chen 2022 |
| --- | --- | --- | --- | --- | --- |
| Study design | Observational, retrospective cohort (uncontrolled) | Observational, prospective cohort (uncontrolled) | Observational, prospective cohort, open | Observational, retrospective cohort (uncontrolled) | Observational, prospective cohort (uncontrolled, open) |
| Place | Japan, 1 center | Netherlands, 1 center | Europe | Colombia, 1 center | China, 1 center |
| Population | Patients> 18 years of age with moderate-severe psoriasis for at least 6 months and compromise of at least 10% of the body surface area | Patients> 18 years with stable psoriasis, in maintenance | Adults with moderate to severe psoriasis, | Patients> 18 years of age with psoriasis, with biological therapy | Patients aged> 18 years with moderate to severe plaque psoriasis (PASI ≥ 10 or BSA ≥ 10%) for at least half a year, with or without psoriatic arthritis. |
| N | 17 (10 de-escalation, 7 standard doses) | 42 (all in de-escalation) | 75 | 467 | 74 |
| Biological * | ADA | ADA, ETN, UST | ETN | ADA, ETN, GUS, ISK, SEK, UST | SEC |
| Duration of treatment prior to de-escalation (weeks) | ≥ 6 months | 6-week trial period | Contraindication, resistance or intolerance to conventional systemic therapies | ≥ 12 meses | ≥ 6 months |
| Eligibility criteria for de-escalation | "Patient preferences" to assign treatment arm | PASI <8 and a test period of 6 weeks before the start of the study was used, in which the PASI score was not allowed to fluctuate ≥3 points | ≥ PASI50 week 12 | Sustained response (DLQI = 0-5; absolute PASI <3 or BSA <1) for at least 12 months. | PASI 90 or higher on a sustained basis at the follow-up visit. |
| De-escalation strategy | Fixed interval:  ADA every 4 weeks | Staggered extension every 12 weeks until:  ADA every 3 or 4 weeks  ETN every week or 2 weeks  UST 45 mg: 45 mg every 16-, 20- or 24-weeks UST 90 mg: 90 mg every 12 weeks 45 mg every 12 weeks | Phase 1: weeks 0-12  Dose reduction: 50 mg once a week (n = 36)  Standard dose 50 mg twice a week (n = 36)  Phase 2: weeks 12-36  Dose reduction: those who received 50 mg twice a week and achieved PASI 50 ("responders") continued 50 mg once a week until week 36 (n = 33) | Reduction of the dose or increase of the interval of application. (unspecified) | Addition of 4 weeks, with a limit of 20 weeks. |
| Monitoring during de-escalation | 36 weeks (after 24 weeks of induction phase) | They were evaluated at weeks 0, 6, 18, 30, 42, 54, 66, and 78. | Weeks 12, 24 y 36 | - | Weeks 4, 12,24 y 36 |
| Treatment results | At week 60, dose reduction versus standard dose: PASI50: 90% versus 100%; PASI75 90% versus 85.7%; PASI90 40% versus 57.1%.  In both groups, all patients who achieved PASI75 at week 24 maintained the response to PASI75 at week 60.  The PASI50 and PASI90 response rate at week 60 was also comparable between both groups (P = 1 and P = 0.63). | Successful interval prolongation at week 78:  ADA: 1/16 (every 3 weeks), 6/16 patients (every 4 weeks). Failure N = 6. Losses to follow-up N = 3.  ETN: 5/16 (every 2 weeks). Failure N = 9. Losses to follow-up N = 1. Consent withdrawn N = 1.  UST 45 mg: 2/9 (every 24 weeks). Failure N = 7. UST 90 mg: failure N = 1.  Successful interval prolongation was defined as PASI ≤ 8 and no self-reported loss of efficacy. | PASI 50 at week 12  50 mg once a week: 75% (27/36)  50 mg twice a week: 92% (33/36). In these patients, the dose of ETN was reduced to 50 mg once a week starting at week 13, with persistence of the PASI 50 response at week 24 in all cases. | 88% of patients in the optimization strategy remain in sustained clinical response after 8 months; 60% of the optimized biological therapy were interleukin 12/23 inhibitors. | 80% of the patients reached PASI 75, while 54% reached PASI 90, and 28% even reached PASI 100 at week 4. In week 12, PASI improved, with 96%, 88% and 76% of patients who achieved PASI 75, PASI 90 and STEP 100, respectively. At week 24, 96%, 86%, and 76% of patients obtained PASI 75, PASI 90, and PASO 100, respectively. In the final follow-up (week 36), the same percentage obtained PASI 75, 84% PASI 90 and 68% PASI 100. |
| Definition of failure | - | Unacceptable increase in disease activity, judged by the patient or PASI> 8 | Loss of PASI 50, at week 36 | - | Failure to achieve PASI 75 in the last follow-up after prolongation of treatment. |
| Time to relapse | - | No data | In the twice a week/once a week group: 30% (10/33) of the patients after an average of 6 weeks of stopping treatment. | 12% (7 patients) relapsed; the incidence rate was 2.2 person months (95% CI: 0.97-4.4), with no significant differences in relapse rates according to biological therapy. | - |
| Retreatment strategy | - | Retreatment with the previous effective dose. | No data |  | Resume treatment for 1 month. |
| Retreatment results | - | No data | No data |  | Patients required a total of 12 injections according to the recommended regimen versus 7.96 (± 1.14) in this study with de-escalation. This represents a reduction in SEC costs with savings of $ 2,141 per patient during the 36 weeks of treatment. |

**Studies on intermittent therapy with biologics in patients with psoriasis**

| Biological agent and study | Dosage | N | Duration: | Response rate (defined by each study) | Adverse events during retreatment |
| --- | --- | --- | --- | --- | --- |
| Adalimumab |  |  |  |  |  |
| Papp, 2011* | Open phase: 40 mg every 2 weeks and withdrawal if PGA ≤2  Retreatment phase: 80 mg Day 1, then 40 mg every 2 weeks | 285 (178 relapses <40 weeks after discontinuation of treatment) | 56 weeks | 69% (PGA ≤1 in 123 of 178 patients who relapsed) | ≥1 AE 47%, infection 26%, AES 3% |
| Certolizumab pegol |  |  |  |  |  |
| Reich, 2012 | 400 mg on Day 1 followed by 200 mg or 400 mg every 2 weeks for 10 weeks | 71 (relapsed patients) | 12 + 12 weeks | 200 mg each 2 weeks: 68% (PASI 75) 35% (PASI 90)  400 mg q2v: 87% (PASI 75) 49% (PASI 90) | ≥1 AE: 45 infection: 26% (200 mg) (200 mg)/16% (400 mg) (400 mg) AES: 0% |
| Etanercept |  |  |  |  |  |
| Moore et al., 2007 | 50 mg twice a week for 12 weeks followed by 50 mg once a week for 12 weeks | 1274 | 12 + 12 weeks | 60% (PGA ≤2) | ≥1 AE: 53% AES: 3% |
| Ortonne et al., 2009 | 25 mg twice a week if relapse (PGA> 2) | 226 (relapsed patients) | 54 weeks | 83% (PGA ≤2) | AES: 9%** |
| Griffiths et al., 2015† | 25 mg twice weekly if relapses (PGA> 2) | 131 (relapsed patients) | 54 weeks | 91% (PGA ≤2) |  |
| Puig et al., 2012 | 25 mg twice a week, 50 mg twice a week or 50 mg once a week | 348 | 12 meses | 64% (PASI 75) 62% (PGA ≤1) |  |
| Luger et al., 2016 | 50 mg twice a week or once a week | 190 | 36 meses | ≥1 AE: 25% Infection: 1% | AES: 4% |
| Infliximab |  |  |  |  |  |
| Menter et al., 2007 | 3 mg/kg every 8 weeks on demand  5 mg/kg every 8 weeks on demand | 148 (3 mg/kg) 149 (5 mg/kg) | 50 weeks | 3 mg/kg every 8 weeks on demand: 25% (PASI 75)  5 mg/kg every 8 weeks on demand: 38% (PASI 75) | 3 mg/kg every 8 weeks on demand: ≥1 AE: 88% Infection: 49% Reaction to infusion: 4% SAE: 3%  5 mg/kg every 8 weeks on demand: ≥1 AE: 83% Infection: 47% Reaction to infusion: 19% SAE: 3% |
| Reich et al., 2013 | 1-4 doses of 5 mg/kg on demand | 219 | 52 weeks | 47% (PASI 75) | ≥1 AE: 71% Infusion related reaction related to infusion: 15% AES 11% |
| Ustekinumab |  |  |  |  |  |
| Leonardi et al., 2008 |  | 195 | 12 weeks | 86% (PASI 75) |  |
| Choi et al., 2018 |  | 16 (12 with 1 retreatment with UST, 3 with 2 retreatments with UST, and 1 with 4 retreatments with UST |  | 67% (PASI 75) | AE or unsatisfactory response: 9% |
| Guselkumab |  |  |  |  |  |
| Reich et al., 2017 | 100 mg on Day 1 and week 4, and every 8 weeks thereafter | 182 | 48 weeks | 37% (PASI 90) | ≥1 AE: 45% Infection: 28% AES: 2%. |
| Brodalumab |  |  |  |  |  |
| Papp et al., 2016 | 140 mg every 2 weeks  210 mg every 2 weeks | 59 84 | 52 weeks | 84% (PGA ≤1) 97% (PGA ≤1) |  |
| Blauvelt et al., 2017 | Initial treatment: 80 mg every 2 weeks or 80 mg every 4 weeks  After relapse: 80 mg every 4 weeks | 157 with starting dose every 2 weeks  176 with starting dose every 4 weeks | 24 weeks | Initial dosing every 2 weeks: 71% (PGA ≤1) 87% (PASI 75)  Initial dose every 4 weeks: 82% (PGA ≤1) 95% (PASI 75) | Initial dosage every 2 weeks: ≥1 AE: 48% Infection: 31% Injection site reaction: 1% AES: 3%  Initial dosage every 4 weeks: ≥1 AE: 50% Infection: 34% Injection site reaction: 3% AES: 1%. |
| Umezawa, et al., 2019 | Induction dosing period:  Initial dose of 160 mg, then 80 mg every 2 weeks  Maintenance dosage Period: 80 mg every 2 weeks  Retreatment period: 80 mg every 2 weeks | 78 | Up to 292 weeks | 100% (PASI 75) 87% (PASI 90) 53% (PASI 100) | During the treatment period: AE: 88 AES: 14%. |
| Secukinumab |  |  |  |  |  |
| Blauvelt et al., 2017 | In case of relapse: 300 mg once a week for 5 weeks followed by 300 mg every 4 weeks | 181 | 16 weeks | 94% (PASI 75) |  |

** Additional efficacy data from a long-term open-label extension of this study were published in Papp* et al.*, 2013, as well as in a post hoc* analysis of health-related quality of life by Gordon et al., 2015.

** Percentage reported for the whole treatment arm on pause (n = 363).

† Post hoc analysis published by Ortonne et al., 2008.

AE: adverse event; PASI: Psoriasis Area Severity Index; PGA: Physician Global Assessment; SAE: serious adverse event.

Source: Al-Hammadi A, J Eur Acad Dermatol Venereol. 2021; 35 (2): 360-7

1. Holey, E. A., Feeley, J. L., Dixon, J. & Whittaker, V. J. An exploration of the use of simple statistics to measure consensus and stability in Delphi studies. *BMC Med. Res. Methodol.* **7**, 52 (2007). [↑](#footnote-ref-1)
